# Supplementary material for: Probing three-dimensional cyclooctatetraene for nucleobase modification in aptamer selection
Source: Commun Chem. 2025 Sep 15;8:276. doi: 10.1038/s42004-025-01629-5 (PMC12436594; doi:10.1038/s42004-025-01629-5)
Supplement: Supplementary file 2 — Supporting Information [file 42004_2025_1629_MOESM2_ESM.pdf]

Probing three-dimensional cyclooctatetraene for nucleobase modification in aptamer selection

Greta Charlotte Dahm,[a] Usman Akhtar,[a,b] Alix Bouvier-Müller,[a] Laura Lim,[a] Fabienne Levi-Acobas,[a] Pierre Nicolas Bizat,[a] Germain Niogret,[a] Julian A. Tanner,[c,d,e] Frédéric Ducongé,[f] and Marcel Hollenstein[a]*

[a] Institut Pasteur, Université Paris Cité, CNRS UMR3523, Department of Structural Biology and Chemistry, Laboratory for Bioorganic Chemistry of Nucleic Acids, 28, rue du Docteur Roux, 75724 Paris Cedex 15, France

[b] Department of Pharmacy, Forman Christian College (A Chartered University), Lahore, Pakistan

[c] School of Biomedical Sciences, LKS Faculty of Medicine, The University of Hong Kong, Hong Kong, China

[d] Advanced Biomedical Instrumentation Centre, Hong Kong Science Park, Shatin, New Territories, Hong Kong, China

[e] Materials Innovation Institute for Life Sciences and Energy (MILES), HKU-SIRI, Shenzhen, Guangdong, 518063, China

[f] CEA, DRF, Institut of biology JACOB, Molecular Imaging Research Center (MIRCen), Université Paris Saclay, CNRS UMR9199, Fontenay aux roses 92335, France

*To whom correspondence should be addressed. E-mail: marcel.hollenstein@pasteur.fr

1. Materials and methods

General information

All reagents and solvents were obtained from commercial sources and were used without further purification. Reaction monitoring was performed by analytical thin layer chromatography (TLC) on glass coated with silica gel 60 F254 from Merck KGaA. Eluted TLCs were visualised under UV light (254 nm) and/or by staining with vanilin or potassium permanganate upon heating. Crude mixtures were purified by flash column chromatography on silica gel 60 (230-400 mesh, 0.040-0.063 mm) purchased from Merck KGaA. Solvents were removed by rotary evaporator below 40˚C and the compounds further dried using high vacuum pumps. HPLC: Purifications procedures employed an Akta pure apparatus from GE Healthcare

Mass Spectrometry

High resolution mass spectrometry (HRMS) was recorded on a Waters Q-tof Micro MS with electrospray ionization (ESI).

MALDI-TOF were conducted on a Bruker ultraflextreme:

-Triphosphates analysis: 9-aminocridine was used as a matrix for triphosphates detection using linear negative mode.

Nuclear Magnetic resonance

Proton (^{1}H), carbon (^{13}C) and phosphorous (^{31}P) nuclear Magnetic Resonance (NMR) spectra were recorded on Bruker UltraShield avance II 500 MHz in the indicated deuterated solvent at a constant temperature of 298 K. Chemical shifts for ^{1}H and ^{13}C spectra are reported on the delta (δ) scale in parts per million (ppm) from low to high field and referenced to residual solvent reference: ^{1}H δ = 7.26 ($CDCl_3$), 2.50 (d^6-DMSO), 4.76 (D_2O), ^{13}C δ = 77.16 ($CDCl_3$), 39.52 (d^6-DMSO). Data are presented as follows: chemical shift, multiplicity (s = singlet, br. s = broad singlet, d = doublet, dd = doublet of doublets, t = triplet, q = quadruplet, m = multiplet), coupling constants (J) expressed in Hz and integration value. Carbon multiplicities were assigned by Distortionless Enhancement by Polarization Transfer (DEPT) experiments. Where required, ^{1}H and ^{13}C signals were assigned by correlation spectroscopy (COSY), Heteronuclear Single Quantum Correlation (HSQC), Heteronuclear Multiple-Bond Correlation spectroscopy (HMBC).

Polyacrylamide (PAGE) gel

Acrylamide/bis(acrylamide)(29:1, 40%) as purchased from Fisher Scientific. PAGE gel analysis was carried out via fluorescence imaging on a GE Healthcare Typhoon Trio.

UV-Vis measurements

UV-Vis analysis were conducted with the UV-Vis CARY 3500 COMPACT PELTIER from Agilent Technologies in 1 mL cuvettes.

HPLC purification

HPLC purification was performed using an ÄKTA pure™ system (GE Healthcare) equipped with an anion exchange high-performance liquid chromatography (HPLC) (Dionex - DNAPac PA200) or semi-preparative reverse phase (RP) HPLC column (Phenomenex Luna - 5μ C18 100Å).

Supporting Protocol 1

Single incorporation of dU^{COTc} via PEX reaction followed by purification and digestion of dsDNA

P5 primer (100 pmol) was annealed with template **T3** (150 pmol) in DNase/RNasefree ultrapure water. This process was carried out by first raising the temperature to 95°C, then gradually reducing it down to room temperature over a period of one hour. The resulting solution was mixed with 1 µL of Vent (*exo*$^{-}$) polymerase, 1 µL of Thermopol buffer and 200 µM of **dU^{COTc}TP 6** and dTTP for positive control, giving a total volume of 10 µL. Next, the combined mixture was left to incubate for a period of 30 minutes at a temperature of 60°C. The resulting products were purified using Monarch DNA Cleanup columns (5 µg), each column processing a maximum capacity of 250 pmol of product. The purified products (around 100 pmol in 10 µL) were then combined with Nucleoside Digestion Mix buffer (2 µL of 10X) and Nucleoside Digestion Mix (1 µL) in a final volume of 20 µL. The reaction mixtures were left to incubate at 37°C for one hour. Finally, the resulting products were subjected to LC-MS analysis without further purification, thus completing the process of verifying the incorporation of a **dU^{COTc}TP 6** into dsDNA.

Protocol for the detection by LC-MS of dU^{COTc} after digestion of dU^{COTc}TP 6 and dsDNA

A solution of digested dsDNA or dUCOBOH was introduced into a ThermoFisher Hypersil Gold aQ chromatography column (100 X 2.1 mm, with a particle size of 1.9 µm), maintained at a temperature of 30°C. Flow rate was set at 0.3 ml/min, and isocratic elution was performed at 1% MeCN in H_2O with 0.1% formic acid for 8 minutes, then at 100% MeCN from the 9th to the 11th minute. In positive ion mode, parent ions were fragmented using a normalized collision energy of 10% in PRM (Parallel Reaction Monitoring) mode. MS2 resolution was set at 17,500 with an AGC target of 2e5, a maximum injection time of 50 ms and an isolation window of 1.0 m/z. The inclusion list contained the following masses: dC (228.1), dA (252.1), dG (268.1), dT (243.1) and nucleoside **dU^{COTc}** (583.2). For detection, chromatograms of ions extracted from the base fragments (± 5 ppm) were used (112.0506 Da for dC; 136.0616 for dA; 152.0565 Da for dG; 127.0501 Da for dT and 467.25 Da for fragmented **dU^{COTc}**. To confirm assignment (fragment ion and retention time), synthetic standards were injected beforehand.

2. Chemical synthesis

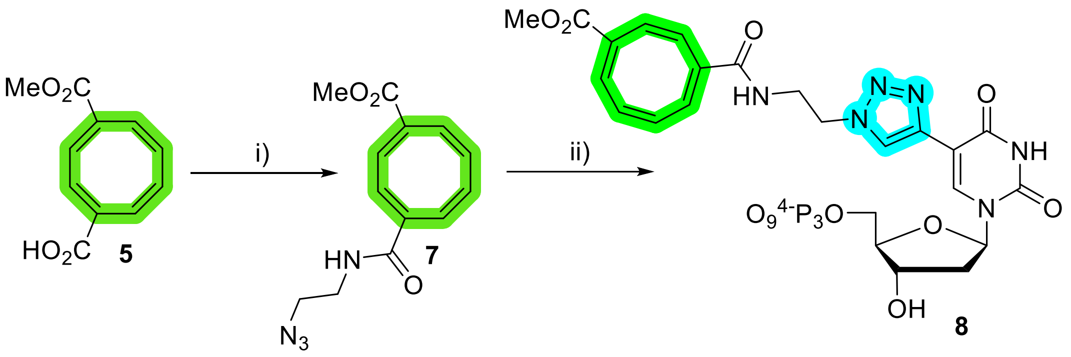

Supplementary Scheme 1. Synthesis of COTc-modified nucleotide **7**. Reagents and conditions: i) DIC, HOBt, DMF, 2-azidoethyl-amine, RT, 48h, 51%; ii) CuI, DIPEA, EdUTP, DMF, MeCN, RT, 3h, 8%.

Synthesis of 4-(methoxycarbonyl)cycloocta-1,3,5,7-tetraene-1-carboxylic acid (**5**)

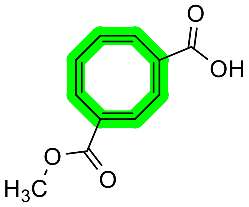

At room temperature and under Ar atmosphere, 4-Methoxycarbonylcubane-1-carboxylic acid (50.0 mg, 0.25 mmol, 1.0 eq.) and bicyclo[2.2.1]heptan-2,5-diene-rhodium(I)chloride dimer (10.0 mg, 0.02 mmol, 0.1 eq.) are suspended in toluene (4 mL) and vigorously stirred for 20 h at 60°C. Progress of the reaction was monitored by TLC (MeOH/DCM 1:4). The crude reaction mixture is purified by column chromatography (MeOH/DCM : from 0 % to 5 % MeOH) after evaporation of the solvents to obtain the title compound **5** (20.5 mg, 41%) as a white solid.

^{1}H-NMR (500 MHz, $CDCl_3$): δ (ppm) 7.17 (dd, J = 4.3, 46.9 Hz, 1H), 7.11 (dd, J = 2.72, 48,5 Hz, 1H), 6.19 (q, J = 11.5 Hz, 1H), 6.06 (m, 3H), 3.78 (d, J = 12.5 Hz, 3H).

^{13}C-NMR (125 MHz, $CDCl_3$): δ (ppm) 176.53, 172.19, 56.19, 55.75, 52.02, 47.48, 47.42.

HR-MS (ESI-negative): *m/z* calculated $[M-H]^-$: 205.0506, found: 205.0500.

Data were in accordance with the literature (*Chem. Eur. J.* **2019**, *25*, 2735-2739).

Synthesis of 5-(methyl 4-((2-(1H-1,2,3-triazol-1-yl)ethyl)carbamoyl)cycloocta-1,3,5,7-tetraene-1-carboxylate)-dUTP **8**

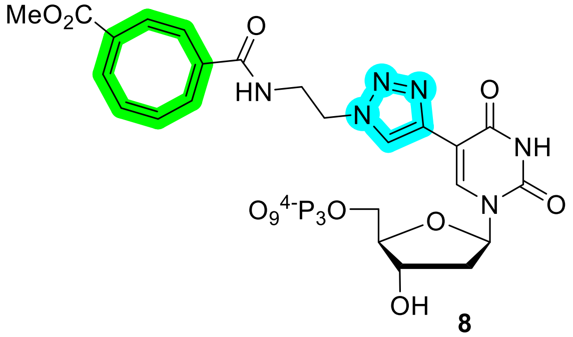

8

All reactants and solvents were degassed. To a solution of CuI (4.65 mg, 0.0244 mmol, 4 eq.) in MeCN (350 µL) and DIPEA (18.25 µL, 0.11 mmol, 36 eq.), methyl-4-((2-azidoethyl)carbamoyl)cycloocta-1,3,5,7-tetraene-1-carboxylate **7** (3.15 mg, 0.0122 mmol, 2 eq.) dissolved in DMF (150 µL) was added. 5-ethynyl-dUTP (3.00 mg, 0.0061 mmol, 1 eq.) dissolved in H_2O (35 µL) was added. The resulting mixture was incubated at 25°C and shacked at 1500 rpm for 3 h. The solvent was removed under reduced pressure and the product was redissolved in water. The crude product was purified via anion exchange HPLC using tetraethylammonium bromide (TEAB, 10 mM) in a gradient from 0% to 100% TEAB (1M). The desired product was obtained as a white powder (0.40 mg, 0.52 µmol, 8%).

HR-MS (ESI-): *m/z* calculated for $C_{24}H_{28}N_6O_{17}P_3^-$ = 765.0729 [M-H]$^-$, found 765.0729.

Synthesis of 5-(methyl 4-((6-oxo-6(prop-2-yn-1-ylamino)hexyl)carbamyol) cycloocta-1,3,5,7-tetraene-1-carboxylate)-dUTP (**dU^{COTc}TP 6**)

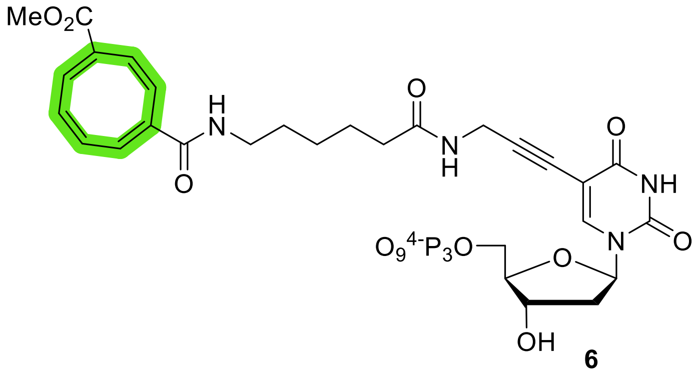

6

Compound **5** (16.5 mg, 80.0 µmol, 3.2 eq.) and HBTU (38.9 mg, 102.5 µmol, 4.1 eq.) are dissolved in DMF (800 µL) at room temperature. DIPEA (16 µL, 95.0 µmol, 3.8 equiv) is added and the reaction mixture is stirred. After 20 min at room temperature, a solution of amino-11-dUTP (15.5 mg, 25 µmol, 1 eq.) in H_2O (100 µL) is added and the reaction mixture is stirred for 17h. After removal of the solvent, the crude reaction mixture was purified via reverse phase HPLC using TEAA (20 mM) in a gradient from 0 % to 50 % MeCN. The titled product is obtained as a clear oil (1.58 mg, 8 %).

^{1}H-NMR (500 MHz, D_2O): δ (ppm) 8.09 (s, 1H), 7.05 (m, 2H), 6.71 (m, 2H), 6.17 (m, 3H), 6.03 (m, 9H), 5.92 (m, 1H), 4.55 (m, 1H), 4.12 (m, 3H), 4.09 (m, 2H), 3.69 (m, 9H), 2.31 (m, 2H), 2.20 (m, 2H), 1.49 (dd, 4H).

^{31}P NMR (202 MHz, D_2O): δ (ppm) -10.99 (d, J = 20.1 Hz), -11.64 (d, J = 20.3 Hz), -23.41 (t, J = 20.0 Hz).

HR-MS (ESI-negative): *m/z* calculated $[M-H]^-$: 821.1243, found: 821.1244.

3. Oligonucleotides

Supplementary Table 1. Sequences used in enzymatic synthesis (shown in the 5' to 3' direction)

Name	Oligonucleotide sequence[a]
Primer **P1**	FAM-GTG CAC CTC GAC CGT AGG
Template **T1**	CGT ACG GTC GAC GCT AGC CCC ATA CTC ATC ACC ATT CAC ATC ACT CAC CTA GCC *ACG TGG AGC TCG GAT CC*
Primer **P2**	CAC TCA CGT CAG TGA CAT GC
Primer **P3**	phos-GTG GTG CGA AAT TTC TGA C
Primer **P4**	biotin-CAC TCA CGT CAG TGA CAT GC
Primer **P5**	FAM-TAC GAC TCA CTA TAG CCT C-3'
Template **T2**	*CAC TCA CGT CAG TGA CAT GCA* TGC CGA TGA CTA GTC GTC ACT AGT GCA CGT AAC GTG CTA *GTC AGA AAT TTC GCA CCA C*[a]
Template **T3**	AGA GGC TAT AGT GAG TCG TA
Library	*CAC TCA CGT CAG TGA CAT GC* N_{30} *G TCA GAA ATT TCG CAC CAC*
Ctrl	*CAC TCA CGT CAG TGA CAT GC* **CTC CTT TCC GCT CGC TCT CTC CTC GCT CCC** *G TCA GAA ATT TCG CAC CAC*
N0	phos-*GTG GTG CGA AAT TTC TGA C* **CGG TAC TCG CTG CGC AGC GCT GCG TAA TGC** *GC ATG TCA CTG ACG TGA GTG*
N1	phos-*GTG GTG CGA AAT TTC TGA C* **ATC CCT GGC GAC CCA GCA TCG TCT GCA CTG** *GC ATG TCA CTG ACG TGA GTG*
N2	phos-*GTG GTG CGA AAT TTC TGA C* **ACT CGG AGG CAC GGC TCA GGA ATT CCG ATT** *GC ATG TCA CTG ACG TGA GTG*
N5	phos-*GTG GTG CGA AAT TTC TGA C* **GAC CAC GAC GCG GGC GCA CAA CTT CGC TTG** *GC ATG TCA CTG ACG TGA GTG*
N6	phos-*GTG GTG CGA AAT TTC TGA C* **GAC GAG ACA CTG TGG GCG TTC TCT TCC GTC** *GC ATG TCA CTG ACG TGA GTG*
N7	phos-*GTG GTG CGA AAT TTC TGA C* **AGT GAC CTG CGC GCA GGT CCA GGA AAT CCT** *GC ATG TCA CTG ACG TGA GTG*
N10	phos-*GTG GTG CGA AAT TTC TGA C* **GGG GAA CGT GCA TGT CTG CAC GGA CGG GGC** *GC ATG TCA CTG ACG TGA GTG*
N15	phos-*GTG GTG CGA AAT TTC TGA C* **CAC GCA AGT CAC TGG GGG CGA GAG GAC CT** *GC ATG TCA CTG ACG TGA GTG*
N42	phos-*GTG GTG CGA AAT TTC TGA C* **GAG AGG TGC CGT CCA CCG ACG AGT ACG GGC** *GC ATG TCA CTG ACG TGA GTG*
N46	phos-*GTG GTG CGA AAT TTC TGA C* **ACG TAG CAT CGA GTC AGC GGG GCA CGC ATT** *GC ATG TCA CTG ACG TGA GTG*

[a] italicized regions represent primer binding regions and bold letters represent reverse complements of identified sequences.

4. Additional figures and images

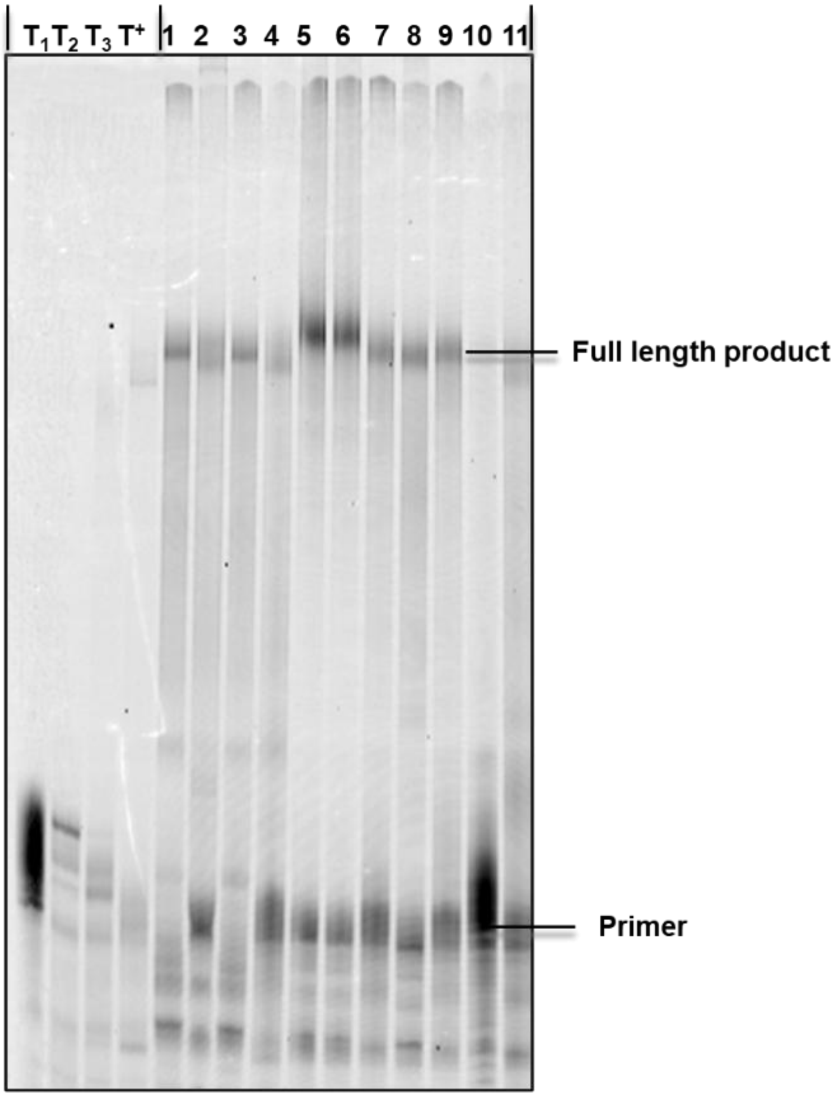

Supplementary Figure 1. Gel analysis (PAGE 20%) of primer extension reactions. The following types and quantities of polymerases were used: lane 1: Phusion (2 U), lane 2: HemoKlem Taq (8 U), lane 3: Q5 (2 U), lane 4: Bst (8 U), lane 5: Taq (5 U), lane 6: Therminator (2 U), lane 7: Vent (*exo*$^{-}$) (2 U), lane 8: Dpo4 (2 U), lane 9: Deep Vent (2 U), lane 10: Kf (*exo*$^{-}$) (5 U). Negative controls: Reaction mixtures containing no polymerase (T_{-1}), only dATP and dGTP (T_{-2}), or dATP, dCTP, and dGTP (T_{-3}) and Taq polymerase. Positive control (T^{+}): with all natural nucleotides and Taq polymerase. All reactions were incubated at adequate reaction temperatures for 1 h in the presence of 200 µM of modified nucleotide **8**.

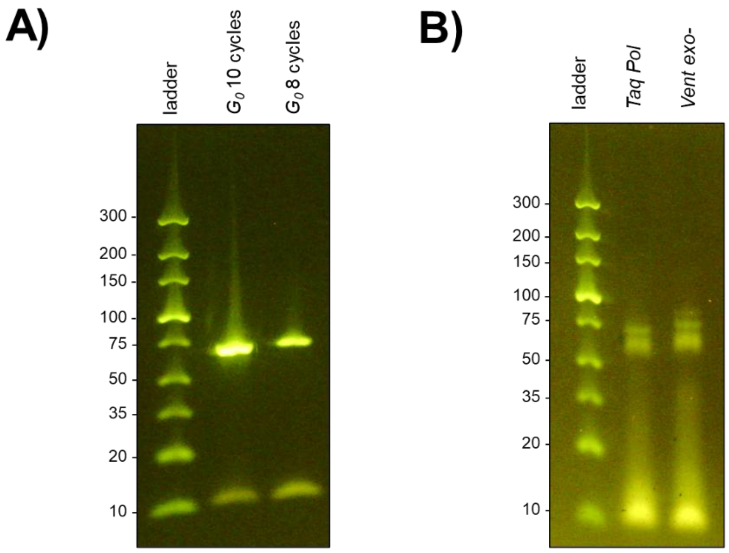

Supplementary Figure 2. A) Agarose gel (4%) analysis of PCR products obtained with a naïve library as a template, primers **P2** and **P3**, and a mixture of natural nucleotides only (i.e. dATP, dCTP, dGTP, and dTTP (all 200 µM)) with either 8 or 10 PCR cycles and Taq polymerase. B) Agarose gel (4%) analysis of PCR products obtained with a naïve library as a template, primers **P2** and **P3**, and Taq or Vent (*exo*$^-$) polymerases and using a mixture of unmodified dNTPs(i.e. dATP, dCTP, and dGTP) and modified nucleotide **8** (all at 200 µM).

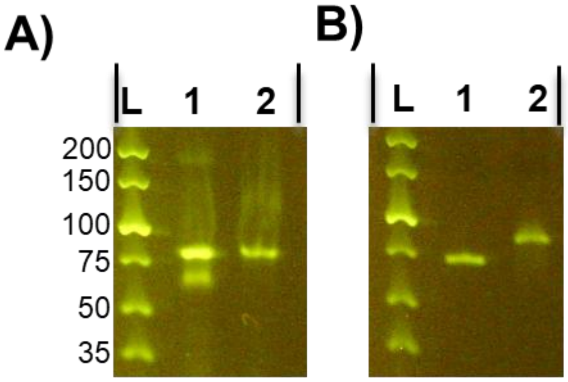

Supplementary Figure 3. A) Agarose gel (4%) analysis of PCR products obtained with a naïve library as a template, primers **P2** and **P3**, and a mixture of dATP, dCTP, dGTP, and **dU^{COTc}TP 6** (all 200 µM). Lane 1: Q5 DNA polymerase, lane 2: Taq; B) Agarose gel (4%) analysis of PCR products obtained with a naïve library as a template, primers **P2** and **P3**, and Taq polymerase. Lane 1: unmodified dNTPs, lane 2: dATP, dCTP, dGTP, and **dU^{COTc}TP 6**.

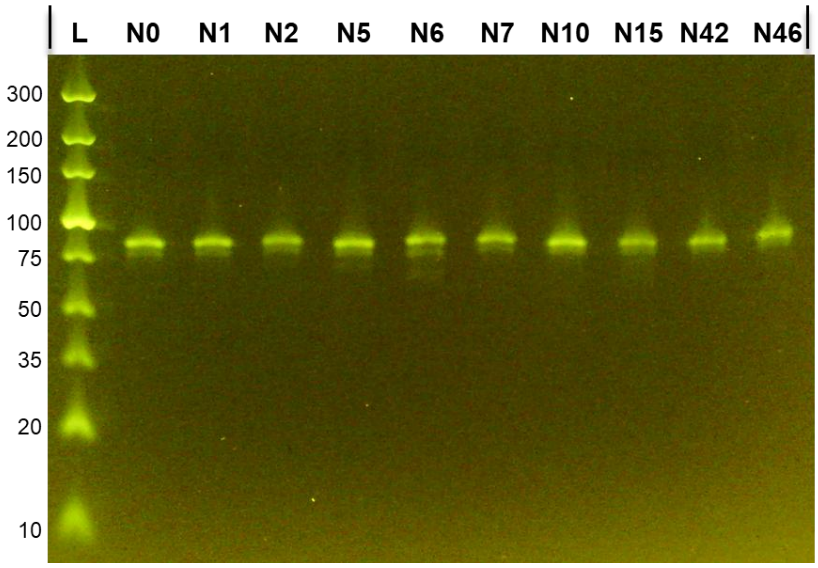

Supplementary Figure 4. Agarose gel (4%) analysis of PCR products obtained individual sequences identified by NGS analysis as templates, primers **P2** and **P3**, and a mixture of dATP, dCTP, dGTP, and **dU^{COTc}TP 6** (all 200 µM).

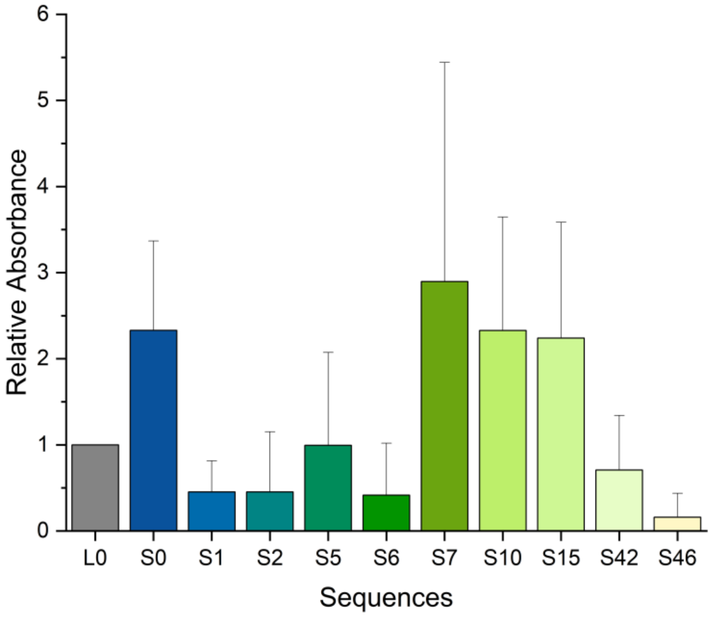

Supplementary Figure 5. Binding studies of the ten aptamer candidates identified by NGS using ELONA assays. Shown is the average and standard deviation of three ELONA replicates.

5. High throughput sequencing

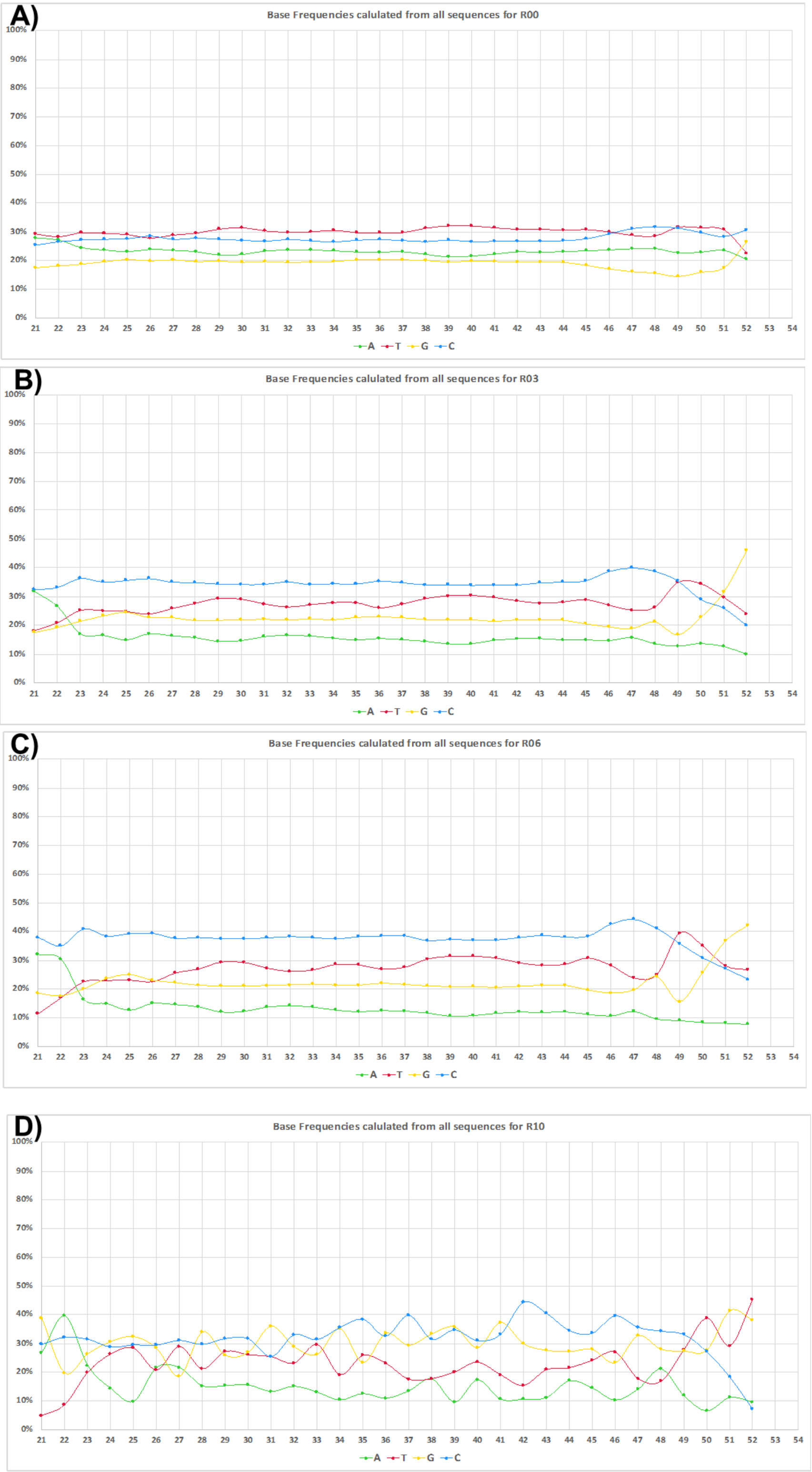

Supplementary Figure 6. Base frequency calculated from all sequences for all sequenced Rounds. Representation A) for the naive library (R00), B) the library of the 3rd round (R03), C) the library 6th round (R06) and D) the library of the 10th round (R10) of the frequencies of A,T,G and C bases at each position of the random part. We tolerated for the analysis 5 deletions or 2 additions in the original 30-nucleotide long random part. Since the forward primer is 20

nucleotide long, these figures represent the base frequencies for all positions between the 21th and the 52th position.

Supplementary Table 2. Size Distribution of random size for each Rounds. This table displays the percentage of sequences having a random region (ie. the size between the two constant primer regions) of a particular size, for each possible random region length (between 0 and 100 nucleotides), for each (naive library (R00), the 3rd round library (R03), the 6th library (R06), and the 10th library (R10). We tolerated for the analysis 5 deletions or 2 additions in the original 30-nucleotide long random part.

Random size (number of base between constant region)	R00	R03	R06	R10
0	0, 163	0, 012	0, 001	0, 000
1	0, 166	0, 050	0, 001	0, 000
2	0, 586	0, 762	0, 132	0, 090
3	1, 311	4, 510	2, 615	6, 328
4	0, 805	1, 975	0, 743	1, 321
5	0, 355	0, 347	0, 052	0, 025
6	0, 216	0, 175	0, 023	0, 010
7	0, 142	0, 047	0, 003	0, 001
8	0, 081	0, 019	0, 003	0, 000
9	0, 056	0, 050	0, 007	0, 010
10	0, 048	0, 081	0, 017	0, 011
11	0, 035	0, 089	0, 026	0, 014
12	0, 036	0, 092	0, 030	0, 017
13	0, 022	0, 048	0, 007	0, 010
14	0, 019	0, 012	0, 006	0, 006
15	0, 018	0, 011	0, 007	0, 030
16	0, 015	0, 016	0, 006	0, 006
17	0, 017	0, 009	0, 005	0, 013
18	0, 016	0, 014	0, 012	0, 028
19	0, 023	0, 018	0, 013	0, 016
20	0, 031	0, 009	0, 026	0, 023
21	0, 019	0, 030	0, 035	0, 027
22	0, 021	0, 035	0, 043	0, 056
23	0, 025	0, 061	0, 058	0, 066
24	0, 030	0, 034	0, 064	0, 066
25	0, 031	0, 069	0, 087	0, 092
26	0, 041	0, 070	0, 100	0, 059
27	0, 091	0, 130	0, 143	0, 115
28	0, 252	0, 408	0, 531	0, 326
29	3, 779	4, 554	5, 411	5, 019
30	88, 647	83, 256	86, 737	84, 113
31	2, 760	2, 791	2, 868	1, 866
32	0, 052	0, 057	0, 093	0, 073
33	0, 006	0, 004	0, 017	0, 027
34	0, 002	0, 006	0, 006	0, 018
35	0, 004	0, 002	0, 005	0, 008
36	0, 005	0, 007	0, 008	0, 013
37	0, 004	0, 006	0, 008	0, 020
38	0, 006	0, 007	0, 007	0, 012
39	0, 002	0, 017	0, 004	0, 015
40	0, 043	0, 084	0, 009	0, 015
41	0, 004	0, 006	0, 008	0, 009
42	0, 008	0, 005	0, 004	0, 006
43	0, 004	0, 004	0, 003	0, 004
44	0, 003	0, 003	0, 003	0, 004
45	0, 000	0, 001	0, 003	0, 005
46	0, 001	0, 001	0, 002	0, 001
47	0, 000	0, 000	0, 002	0, 001
48	0, 000	0, 003	0, 001	0, 000
49	0, 001	0, 000	0, 000	0, 003
50	0, 004	0, 002	0, 001	0, 000

**51**	0, 000	0, 000	0, 001	0, 002
**52**	0, 000	0, 000	0, 000	0, 000
**53**	0, 000	0, 000	0, 001	0, 000
**54**	0, 000	0, 000	0, 000	0, 000
**55**	0, 000	0, 000	0, 000	0, 000
**56**	0, 000	0, 000	0, 000	0, 000
**57**	0, 000	0, 000	0, 000	0, 000
**58**	0, 000	0, 000	0, 000	0, 000
**59**	0, 000	0, 000	0, 000	0, 000
**60**	0, 000	0, 000	0, 000	0, 000
**61**	0, 000	0, 000	0, 000	0, 000
**62**	0, 000	0, 000	0, 000	0, 000
**63**	0, 000	0, 000	0, 000	0, 000
**64**	0, 000	0, 000	0, 000	0, 000
**65**	0, 000	0, 000	0, 000	0, 000
**66**	0, 000	0, 000	0, 000	0, 000
**67**	0, 000	0, 000	0, 000	0, 000
**68**	0, 000	0, 000	0, 000	0, 000
**69**	0, 000	0, 000	0, 000	0, 000
**70**	0, 000	0, 000	0, 000	0, 000
**71**	0, 000	0, 000	0, 000	0, 000
**72**	0, 000	0, 000	0, 000	0, 000
**73**	0, 000	0, 000	0, 000	0, 000
**74**	0, 000	0, 000	0, 000	0, 000
**75**	0, 000	0, 000	0, 000	0, 000
**76**	0, 000	0, 000	0, 000	0, 000
**77**	0, 000	0, 000	0, 000	0, 000
**78**	0, 000	0, 000	0, 000	0, 000
**79**	0, 000	0, 000	0, 000	0, 000
**80**	0, 000	0, 000	0, 000	0, 000
**81**	0, 000	0, 000	0, 000	0, 000
**82**	0, 000	0, 000	0, 000	0, 000
**83**	0, 000	0, 000	0, 000	0, 000
**84**	0, 000	0, 000	0, 000	0, 000
**85**	0, 000	0, 000	0, 000	0, 000
**86**	0, 000	0, 000	0, 000	0, 000
**87**	0, 000	0, 000	0, 000	0, 000
**88**	0, 000	0, 000	0, 000	0, 000
**89**	0, 000	0, 000	0, 000	0, 000
**90**	0, 000	0, 000	0, 000	0, 000
**91**	0, 000	0, 000	0, 000	0, 000
**92**	0, 000	0, 000	0, 000	0, 000
**93**	0, 000	0, 000	0, 000	0, 000
**94**	0, 000	0, 000	0, 000	0, 000
**95**	0, 000	0, 000	0, 000	0, 000
**96**	0, 000	0, 000	0, 000	0, 000
**97**	0, 000	0, 000	0, 000	0, 000
**98**	0, 000	0, 000	0, 000	0, 000
**99**	0, 000	0, 000	0, 000	0, 000

Supplementary Table 3. Evolution of the percentage of each cluster in the library inside the different rounds of SELEX. This table presents for each cluster, its lead sequence, and the percentage of presence of the whole cluster inside each library. For instance, all the sequences

belonging to the cluster 0 represent 0,0201% of the library of round 6, and 5,2695 % of the library of round 10. Clusters colored in light pink are clusters selected for the binding tests.

	Number of reads per library after quality filtering:		90 991	88 766	94 630	111 131
	**Lead Sequence of the cluster**	Cluster	Naive library	Round 3	Round 6	Round 10
Lead 0	GCATTACGCAGCGCTGCGCAGCGAGTACCG	0	0,0022	0,0000	0,0201	5,2695
Lead 1	CAGTGCAGACGATGCTGGGTCGCCAGGGAT	1	0,0033	0,0000	0,0222	3,2835
Lead 2	AATCGGAATTCCTGAGCCGTGCCTCCGAGT	2	0,0022	0,0000	0,0127	2,2667
Lead 3	GGAATTGGGCTGAGCCGAGGTCTCTCGAGT	3	0,0011	0,0000	0,0074	1,7862
Lead 4	CACGGATCTGGGTCGCCAGGGCCGCATCCT	4	0,0011	0,0000	0,0180	1,9706
Lead 5	CAAGCGAAGTTGTGCGCCCGCGTCGTGGTC	5	0,0000	0,0000	0,0180	1,5342
Lead 6	GACGGAAGAGAACGCCCACAGTGTCTCGTC	6	0,0000	0,0000	0,0095	1,1536
Lead 7	AGGATTTCCTGGACCTGCGCGCAGGTCACT	7	0,0022	0,0000	0,0074	0,9583
Lead 8	AGCGGTCTGGGTCGCCAGAACTCGCACGTG	8	0,0000	0,0000	0,0095	0,9862
Lead 9	CGGGCCGAGATTCTTGAGCCGCCAACGAGT	9	0,0022	0,0000	0,0042	0,8108
Lead 10	GCCCCGTCCGTGCAGACATGCACGTTCCCC	10	0,0011	0,0000	0,0159	0,7928
Lead 11	GCGGGACTCTGCCGAACCGGTCCGTGTGTC	11	0,0011	0,0011	0,0137	0,7352
Lead 12	CATGCGATCCTTTCTGGGCCGGACTCGAGT	12	0,0000	0,0000	0,0095	0,7028
Lead 13	AGCAGCTTACCTCGGCTCTGGGTCGCCAGG	13	0,0000	0,0000	0,0095	0,6587
Lead 14	ATTTCCTCCAGGCAGGGCCTGAGTACGGAC	14	0,0000	0,0000	0,0116	0,4949
Lead 15	AGGTCCTCTCGCCCCCAGTGACTTGCGTG	15	0,0000	0,0000	0,0021	0,4760
Lead 16	GCTCTGAGCCGAGGGAATCGTCGCTCGTGT	16	0,0000	0,0000	0,0095	0,4553
Lead 17	GCCTCGTCCGCCCGTATCTAGGCGGTCCCC	17	0,0000	0,0000	0,0106	0,4229
Lead 18	GACGTCTGTGGTCGCTCGGGCGCGCATGTG	18	0,0000	0,0000	0,0085	0,4211
Lead 19	GCTGTGACTGATGGACCAGGGCTGAGTGCC	19	0,0000	0,0000	0,0095	0,4004
Lead 20	GCAGAATTTCTGAGCGAGCACGGGCCCTGT	20	0,0000	0,0000	0,0063	0,3590
Lead 21	GAGACATTTCTGGGCCGGGTCTTGCCGTGT	21	0,0000	0,0000	0,0042	0,3257
Lead 22	GATTTCCTGGAGACTCGTCTCACTGGCGAC	22	0,0000	0,0000	0,0063	0,3095
Lead 23	CCGCCATGCGACTGACATTGGGTCGCCAGG	23	0,0011	0,0000	0,0021	0,3068
Lead 24	GCCACGTAACGGGCCTCCACCCGTTTCCCC	24	0,0011	0,0000	0,0021	0,2960
Lead 25	GAGCTGAGCAACCCGCTATCACGTGGCCTT	25	0,0011	0,0011	0,0053	0,2897
Lead 26	GGCAATTGGTGAGCAGAGACGCCCTCGAGT	26	0,0000	0,0000	0,0021	0,2664
Lead 27	AGAGCGCAGTTTAGTGGACCGCACACGAGT	27	0,0000	0,0000	0,0032	0,2888
Lead 28	GAGTTCCTGGAGCACGCCTCACGACCATCG	28	0,0000	0,0000	0,0085	0,2439
Lead 29	AGCGTTTCGGAGCCGATGCCCACTTCGAGT	29	0,0000	0,0000	0,0085	0,2430
Lead 30	ACGTTCTCGACCGGTCCGAGTGCGGGATAC	30	0,0000	0,0000	0,0042	0,2349
Lead 31	ACTCGGCCATCGTCGGTTGGCGGGCACGTG	31	0,0011	0,0000	0,0032	0,2331
Lead 32	GAATTGTCGCCCGGAGCTTGTGCTCGCGTG	32	0,0000	0,0000	0,0032	0,2268
Lead 33	CCCCCCCTAGTGTTCTGAGCCGAACCGAGT	33	0,0000	0,0011	0,0032	0,4202
Lead 34	GATGTGTGGGTCGCCAGCGCGCATGTAGGT	34	0,0000	0,0000	0,0011	0,3176
Lead 35	CATGTGGGTCGCCACGTGGCTGCTAGCTCT	35	0,0000	0,0000	0,0042	0,2124
Lead 36	GCACCCCGGTATTAGGCAGCACGCCTCCCC	36	0,0000	0,0000	0,0032	0,2124
Lead 37	ACTGCGGGTAATTCCTGAGCCGCTCCGAGT	37	0,0000	0,0011	0,0021	0,2079
Lead 38	AGCGGCATATGTTTCTGAGCGGGCCCCAGT	38	0,0000	0,0000	0,0032	0,2052
Lead 39	GGCGCACGAATCGCTTGGGTCGCCAGGCGA	39	0,0000	0,0000	0,0021	0,1998
Lead 40	GCTCGTAGGGCGACGAAGCAGCCCTTCCCC	40	0,0011	0,0000	0,0053	0,1989
Lead 41	GACGTTTGAGCCAACCTCCTCGACCACTCG	41	0,0000	0,0000	0,0032	0,1980
Lead 42	GCCCGTACTCGTCGGTGGACGGCACCTCTC	42	0,0000	0,0000	0,0053	0,1971
Lead 43	GAATCGACTTGATTGGCCGACACGAGTGCA	43	0,0000	0,0000	0,0011	0,1944
Lead 44	GCATTTCCTGAGCCGATGCTTCTGCCGAGT	44	0,0000	0,0011	0,0011	0,1800
Lead 45	CAGTCGAATGTTCTGGGCCGCCAACGTGT	45	0,0000	0,0011	0,0032	0,2178
Lead 46	AATGCGTGCCCCGCTGACTCGATGCTACGT	46	0,0000	0,0000	0,0042	0,1755
Lead 47	GAGAGGACCACAGAGGTTGTACTGCTCGTG	47	0,0011	0,0011	0,0053	0,3590
Lead 48	GAACTTCCTCGACCCGCTGGTCATTGCCAC	48	0,0000	0,0000	0,0011	0,1728
Lead 49	CAACGAGATCTGGGTCGCCAGCTCTCCGTG	49	0,0000	0,0000	0,0021	0,2097
Lead 50	GTTCTGTAGCCGTCCGCCTGCCGCACGAGT	50	0,0000	0,0000	0,0021	0,1620
Lead 51	GCTCGCTCCGTTTTCTGGGCCGCTCCGAGT	51	0,0000	0,0011	0,0021	0,1539
Lead 52	GACCCGGTTCTGCTAGCGGTCCCCAGTGTA	52	0,0000	0,0000	0,0021	0,1692
Lead 53	GAATCTCCGTCCCGCCCGGCTGCGGGTCCC	53	0,0000	0,0000	0,0106	0,1494
Lead 54	GGAGAGCGATATTGCTGAGCCATGTTGCGT	54	0,0000	0,0000	0,0032	0,1494
Lead 55	ATTGGGTCGCCAGATGCATACCCCGCCGTG	55	0,0000	0,0000	0,0000	0,1422
Lead 56	CCTGACCCGCATGGCTCCGAAGCACTCATG	56	0,0000	0,0000	0,0032	0,1764
Lead 57	GAGGGATCCCAGGCTTGCGCACGCTATTC	57	0,0000	0,0000	0,0021	0,1512
Lead 58	GAGAGCTCTTACCGACCCGCTACGTGGCTC	58	0,0000	0,0000	0,0032	0,1305
Lead 59	GAGATCTGTCTGAGCCGGAAGCTACCGTGT	59	0,0000	0,0000	0,0063	0,1269
Lead 60	GCAGTCCCTGTTTCCTGAGCGGTCCCCAGT	60	0,0000	0,0000	0,0021	0,1242
Lead 61	ACTGTGCACCCGGCTCCGTGGCTGTCCAGG	61	0,0000	0,0000	0,0063	0,1224
Lead 62	ACGCAATCTGGGTCGCCAGCATTGGGTCCC	62	0,0000	0,0000	0,0032	0,1197
Lead 63	AGATTTCATTACCGGCCCACCCAGGCACGA	63	0,0000	0,0000	0,0042	0,1296
Lead 64	CCACGAAGTTTCGGAGCGGTCCCATCCTGT	64	0,0000	0,0000	0,0021	0,1188
Lead 65	AACCAGCCCGACACGTTGGGTCGCCACCGA	65	0,0000	0,0000	0,0063	0,1161
Lead 66	CACCTCGGATATTGCTGAGCGGATCCCAGT	66	0,0000	0,0000	0,0000	0,1116
Lead 67	GAGCGTCGATTAGGTGTCACGCACCTCCCC	67	0,0000	0,0000	0,0032	0,1098
Lead 68	ACGGCGCATTTCCTGGACCAACGGTCACCT	68	0,0011	0,0011	0,0000	0,1089
Lead 69	AGGAATTGGGGAGCCGGCGATCCGTCGAGT	69	0,0000	0,0000	0,0074	0,1494
Lead 70	GCCCCGTCTCGCAGAGTATGTTCTGCCCTC	70	0,0000	0,0000	0,0021	0,1062
Lead 71	CCAGCGCTCGTCCTCGTACCAGAGGTCCCC	71	0,0000	0,0000	0,0074	0,1053
Lead 72	ATACTCCGCTAGGCGGTTGGGTCGCCAGCC	72	0,0000	0,0000	0,0000	0,1044
Lead 73	GCCCGACCATATTCTTGGCCCCCGTCGGTG	73	0,0000	0,0000	0,0021	0,1026
Lead 74	CCCTGGTGGTCGCTCCAGGGCCTACTCCCC	74	0,0000	0,0000	0,0000	0,1017
Lead 75	GCCACGTCCCGACTCACTAGGGGAGGTCCC	75	0,0011	0,0000	0,0011	0,1017
Lead 76	GAGCATTTCATGAGCCGCTATCGGGCGAGT	76	0,0000	0,0000	0,0000	0,1008
Lead 77	CAGGATCGCTTGGGTCGCCAGCGGGCCAAG	77	0,0000	0,0000	0,0042	0,0999
Lead 78	CATACCCATTCGCTCGATCTGGGTCGCCAG	78	0,0000	0,0000	0,0021	0,0981
Lead 79	ACGGGATGGCTTGCCCCCGGGGTCTCGGTG	79	0,0000	0,0000	0,0042	0,0963
Lead 80	TGGGGTCTGGGTCGCCAGAGGCCGCCAGTG	80	0,0011	0,0000	0,0000	0,0963
Lead 81	CCGGATGCGTCTCGTCCACGAAGGGGTCCC	81	0,0000	0,0000	0,0021	0,0945
Lead 82	AACGTATGGGTCGCCAGACGAGCGTCACCT	82	0,0011	0,0000	0,0000	0,0927
Lead 83	AATGGCCGCGGTTCCTTGTCGATGGAGCCC	83	0,0000	0,0000	0,0000	0,1125
Lead 84	TCGATCGGATGCTTTATCCATGCTTTTCGA	84	0,0550	0,0676	0,0898	0,0747

Lead 85	ATATCGCCGCGGCACCGACTATTGGCGAAT	85	0,0011	0,0000	0,0063	0,0882
Lead 86	CACTCGTTGGGTCGCCAGGCAAGTGGCTCG	86	0,0000	0,0000	0,0053	0,0882
Lead 87	CACGCATCTGAGAGCCGAGTAGTCGAGTGT	87	0,0011	0,0011	0,0095	0,0864
Lead 88	GAGTGTCCACTTGCGTAGCTGTTCTGGCCT	88	0,0000	0,0000	0,0021	0,1008
Lead 89	GCGAGCAAGTCCTCTCATCTAGGGAGTGTG	89	0,0011	0,0000	0,0032	0,0864
Lead 90	CAGTGCTCGTATCGCAACTTGGGTCGCCAG	90	0,0000	0,0011	0,0032	0,1152
Lead 91	GATGCCGTGTCTGTCTGAGCCGCTGCGAGT	91	0,0000	0,0000	0,0032	0,0846
Lead 92	GAGCTTATTAGCACCCCGCTGGAGCTCCCG	92	0,0000	0,0011	0,0053	0,1890
Lead 93	GCTCGTCCCGGGAGTACTGTGCCCGGTCCG	93	0,0000	0,0000	0,0011	0,0837
Lead 94	CCTGTCGATGTCGATGGCACGGGCGCACCT	94	0,0000	0,0000	0,0011	0,0828
Lead 95	GCATTTGTGAGCCGGCATCTCGCATCGTGT	95	0,0000	0,0000	0,0000	0,0819
Lead 96	ACGCCAACGGTCGCATTCTGTCGATGCTAC	96	0,0000	0,0000	0,0000	0,0810
Lead 97	CGTCCTCTCGCACCCAGTGACGGCACTGGA	97	0,0000	0,0000	0,0000	0,0810
Lead 98	GAATGCCTATGTTTGTGAGCCGATCCGAGT	98	0,0000	0,0000	0,0011	0,0801
Lead 99	TGGTCTGCTGGTAGGACCGCTCCGAGTGCC	99	0,0000	0,0000	0,0000	0,0792
Lead 100	CGCGACGGTACGCTGGACGATACCGCTTGT	100	0,0000	0,0000	0,0021	0,0774
Lead 101	GACGGCTACGGGATTGGGTCGCCAGTCCCC	101	0,0000	0,0000	0,0021	0,0774
Lead 102	AACACGCGTCCCCTGCTTGCGCAGCTGTTC	102	0,0000	0,0000	0,0000	0,0765
Lead 103	GCTAATTCTCTGGGCCGTCGCAGTACGAGT	103	0,0000	0,0000	0,0000	0,0900
Lead 104	GTCCCGTCCGGCACACACGAGCCGGTTCCC	104	0,0000	0,0000	0,0021	0,0747
Lead 105	ACGGCAGGTTGTAGTGCTGCCGGGCCGCCA	105	0,0000	0,0000	0,0000	0,0873
Lead 106	AGTGGTCGTCGGTCCGCTGCGGGTGTCCGT	106	0,0000	0,0000	0,0021	0,0918
Lead 107	TAGGATCTGGGTCGCCACGGTCCTCCCCCG	107	0,0000	0,0000	0,0021	0,0729
Lead 108	GACGCAAATTCGTGAGCCGCGGTAGCGTGT	108	0,0000	0,0000	0,0021	0,0711
Lead 109	GACGTACGGCCGATACGCGCACTCCACCTT	109	0,0011	0,0000	0,0011	0,0711
Lead 110	AGATTCTGAGCCGATGTTGTACCCCGAGT	110	0,0000	0,0000	0,0021	0,1377
Lead 111	GGCCCGTCCGGGGACTTCCTCCCCGGTCCC	111	0,0011	0,0000	0,0042	0,0702
Lead 112	CAGACCGCACTTCTGAGCCGATCTTCGAGT	112	0,0000	0,0000	0,0000	0,0684
Lead 113	GCCCCGTAGTCTCGGGTACCCGGACTCCC	113	0,0000	0,0000	0,0063	0,0684
Lead 114	CACTCCCGCTGACCTATGGGTCGCCAGCAG	114	0,0000	0,0000	0,0000	0,0675
Lead 115	CCATACGTGCACGGCCTGATTGGGTCGCCA	115	0,0000	0,0000	0,0000	0,0657
Lead 116	CGGTCTGGCTGGAATTCTGGACGATGCTCC	116	0,0000	0,0000	0,0042	0,0657
Lead 117	GCTATCTGAGCCAACGGCCCTCCCAGTGTG	117	0,0000	0,0000	0,0032	0,0657
Lead 118	ACGCCGGCTGCCTCCTCTGTACGATGCTAC	118	0,0000	0,0000	0,0000	0,1017
Lead 119	ACGTTGGGTCGCCAAGGGCATTCCCTCCCG	119	0,0011	0,0000	0,0021	0,0648
Lead 120	AGGGGCAGGCCGTTTGGGTCGCCGAACGAC	120	0,0000	0,0000	0,0011	0,0648
Lead 121	GAGTACTGTCTCTGGGTCGCCAGAGGTCCG	121	0,0000	0,0000	0,0053	0,0648
Lead 122	GTACGGACTCACCCTGCTGGGTCGCCAGAG	122	0,0000	0,0000	0,0021	0,0810
Lead 123	GTCGACGAGCTTGGGTCGCCAGCCGGCAGA	123	0,0000	0,0000	0,0000	0,0648
Lead 124	AAATTATCCTGGGCCGAGGAGTCCTCGAGT	124	0,0000	0,0000	0,0000	0,0900
Lead 125	GCTCGTCATGTTCTTGACCACGGGTCGGTG	125	0,0000	0,0000	0,0011	0,0639
Lead 126	AGAGGGCGCTTCTCGTCCGGTACGGTCTCC	126	0,0000	0,0000	0,0106	0,0630
Lead 127	GGCGCCATACGTGGTTCTGCCAGCGCTCCT	127	0,0000	0,0000	0,0011	0,0630
Lead 128	CCGCAGCGTGTGGTCGCTCACCCAGGCCCTT	128	0,0000	0,0000	0,0000	0,0621
Lead 129	CGCAGCAAGAGGTGCGCCCGTCATCTCCCC	129	0,0000	0,0000	0,0011	0,0621
Lead 130	GAGCCACCATCCTCGTCGGTGGATGGGCGT	130	0,0000	0,0000	0,0000	0,0621
Lead 131	GTAGTTCTCTGTCAGCCGCCACCCGAGTGT	131	0,0000	0,0000	0,0000	0,0621
Lead 132	AAGTCCCCATCGTTCTGGACCGACCCGAGT	132	0,0000	0,0000	0,0032	0,0612
Lead 133	CAGGCTCCTTGTCGATGGCAGCCTAGGGTG	133	0,0000	0,0000	0,0032	0,0612
Lead 134	GACTACTGAGCCAACCGCTATACGAGTGTG	134	0,0000	0,0000	0,0032	0,0612
Lead 135	GAGGTTTCTCGAGCGGCACTAGGTGCCAGT	135	0,0000	0,0000	0,0021	0,0612
Lead 136	CCAGTCCGGGCTCACTTGCGTACGCTGTTC	136	0,0000	0,0000	0,0063	0,0603
Lead 137	GCTCACTGCTATGGACCGCCACGAGTGCCA	137	0,0000	0,0000	0,0011	0,0603
Lead 138	GCCTCGTTCCCTAGCCGAATCGCGGGCCCA	138	0,0000	0,0000	0,0021	0,0594
Lead 139	GCTCGATGTCGATGGGCGCCACTATGCGTG	139	0,0000	0,0000	0,0042	0,0594
Lead 140	TACCGACGCAATTGACCTTGGGTCGCCAGG	140	0,0000	0,0000	0,0011	0,0594
Lead 141	AATCGGGGGCTGGACTAGTCGTCGGTCTAG	141	0,0000	0,0000	0,0011	0,0585
Lead 142	GAGCGCTCTCTCTTCTGCTTGGGTCGCCAG	142	0,0000	0,0000	0,0011	0,0585
Lead 143	GACCCACCTGGGTCAGTGAGCCCTAGGCTC	143	0,0000	0,0000	0,0021	0,0576
Lead 144	GACGACCTCGTTTCTGAGCCAGCATTGTGT	144	0,0000	0,0000	0,0000	0,0576
Lead 145	GATGTATGAGCCGGGTCCGCCGCCCGTGT	145	0,0000	0,0000	0,0042	0,0576
Lead 146	CAGGCACCTTGGGTCGCCACAGGTCCCCCC	146	0,0000	0,0000	0,0021	0,0567
Lead 147	GAACTAGGGTTGGGTCGCCAGCACTACGTG	147	0,0000	0,0000	0,0011	0,0567
Lead 148	GAGAGTAGTTGTTTCTGGACGGGTCCCAGT	148	0,0000	0,0000	0,0000	0,0756
Lead 149	GTGCTAGTGAGCGAGCCGTTTCCGAGTGTC	149	0,0000	0,0000	0,0000	0,0567
Lead 150	AGGTCTGGGTCGCCAGACCACGCCGCCACT	150	0,0000	0,0000	0,0021	0,0558
Lead 151	GCCCACAGGGGTTTGCGTCCGCTGTTCTGG	151	0,0000	0,0000	0,0032	0,0558
Lead 152	ACCTAGGGGAAGCGCTCGTCCGGAGGTCTC	152	0,0000	0,0000	0,0011	0,0549
Lead 153	GAAGGGTTCTGGTTGGCCGTCTCGAGTGTA	153	0,0000	0,0000	0,0000	0,0549
Lead 154	GACCACCAGGGGATTTCCGCCCGTTCCGTG	154	0,0000	0,0000	0,0011	0,0666
Lead 155	TAGTTCTGAGCCGGTGGACCCTGGCCGTGT	155	0,0000	0,0000	0,0063	0,0540
Lead 156	ACGACGTGCAGGCGAAGGTTGCTGTCGTCG	156	0,0000	0,0000	0,0011	0,0531
Lead 157	CATCAGCTCTGGGTCGCCGGAGCAGCTCTA	157	0,0000	0,0000	0,0011	0,0531
Lead 158	GGGGAGGAGATGTCGATTGAGTGCCCCGTG	158	0,0011	0,0000	0,0000	0,0522
Lead 159	ATTGGGTCGCCAGTGCACTTGCGCGATGTG	159	0,0000	0,0000	0,0011	0,0513
Lead 160	CGCGCAATCGTAGTTGGGTCGCCAGCTCAC	160	0,0000	0,0000	0,0000	0,0513
Lead 161	GAACTCACGCATCGGGCTTGGGTCGCCACA	161	0,0000	0,0000	0,0053	0,0954
Lead 162	GAATATTGCAGAGCCGGCCAGATCCCGAGT	162	0,0000	0,0011	0,0042	0,0513
Lead 163	TAGTGACCGCTCCGAGTGCACGGGCAGGGT	163	0,0000	0,0000	0,0011	0,0504
Lead 164	AATGCGGATGCTGGACACCGAGTCGAGTCT	164	0,0000	0,0000	0,0032	0,0495
Lead 165	CATGGAGGCATCTTCTGGCTGGATGCTACCT	165	0,0000	0,0000	0,0011	0,0495
Lead 166	CATGTCCTCTCACCCTTGCGCGAGTGTGTG	166	0,0000	0,0000	0,0000	0,0495
Lead 167	CCGGTACTTCTGGCAGTTGGGTCGCCAGCC	167	0,0000	0,0000	0,0011	0,0495
Lead 168	CAGTGACCCCGCGTCCTCTCACCATCCCTC	168	0,0000	0,0000	0,0053	0,0486
Lead 169	TACCCCGAGATTTCTTGAGCGGCAGCCAGT	169	0,0000	0,0000	0,0000	0,0486
Lead 170	AACTGGGGCTCTGGACACGGTTCCCAGTCT	170	0,0000	0,0000	0,0011	0,0459

Lead 171	GAGGAATTGTCCCTGAGCGATTGTCGATTC	171	0,0000	0,0000	0,0000	0,0459
Lead 172	ATGCCATCGTTCTCTGAGCCGCCAGCGAGT	172	0,0000	0,0000	0,0053	0,0594
Lead 173	CCTCCTGCACGAACTTCGTACCGATGCTAC	173	0,0000	0,0000	0,0021	0,0450
Lead 174	GATGTCGCTCCAGTCGTCGGTCTGAGCGTC	174	0,0000	0,0000	0,0000	0,0576
Lead 175	GCCCCGTCACTGCGGCCAACGCGGTTCTCC	175	0,0000	0,0000	0,0011	0,0450
Lead 176	ACTGGAGTTGGGTCGCCAACTGCACGGGTG	176	0,0000	0,0000	0,0032	0,0774
Lead 177	ACCAATTATTGCGTTGGTTTTGTGATAAAA	177	0,0220	0,0192	0,0433	0,0306
Lead 178	CGGCGATGTCCCTCGGGATTCTGCCCTAGG	178	0,0000	0,0000	0,0074	0,0432
Lead 179	AGTTGGGCTCTAGACTCGTGGGTCGCCAGA	179	0,0000	0,0000	0,0032	0,0423
Lead 180	CACGGAATTTCTGTAGCCGAGCATTCGTGG	180	0,0000	0,0000	0,0000	0,0423
Lead 181	CACGGGACCGACCAGTTGGGTCGCCAGCTA	181	0,0000	0,0000	0,0032	0,0558
Lead 182	GACTGGTTGCGGGGCTCATCTCCGCTATTC	182	0,0000	0,0000	0,0011	0,0423
Lead 183	AGAGCCCGATATTCTCGAACCGTTCCGAGT	183	0,0000	0,0000	0,0000	0,0414
Lead 184	CAAGTTTCTGAGCGAGTACCTCCCCTCTGT	184	0,0000	0,0000	0,0011	0,0414
Lead 185	CTAGTGCAATTATCCTGAGCCGCGCCGAGT	185	0,0000	0,0000	0,0032	0,0414
Lead 186	GAATTTTGGTGGCGAACGGCTCCCAGTGTC	186	0,0000	0,0000	0,0000	0,0414
Lead 187	TATCTCCCTCCGCTGACTTGGGTCGCCACT	187	0,0000	0,0000	0,0021	0,0666
Lead 188	CAAGCGTGGATTGTCGATTCCCGCCCACCT	188	0,0000	0,0000	0,0011	0,0405
Lead 189	CACCGGACAGGGGTTGGGTCGCCAATACTT	189	0,0000	0,0000	0,0021	0,0405
Lead 190	CATCTAGTCGCAATTGTCGCCGGCTCCCTG	190	0,0000	0,0011	0,0011	0,0405
Lead 191	CTGAGCCGTACGCCCGCCGATCCAGTGGAG	191	0,0000	0,0000	0,0000	0,0405
Lead 192	GACGCCAATCTTGTGAGCCGGCACTCGAGT	192	0,0000	0,0000	0,0042	0,0522
Lead 193	GAGATCTGGGTCGCCAGGCGCAGTGCGTTT	193	0,0000	0,0000	0,0063	0,0405
Lead 194	GATTCATGGACCGCCGGTCACTGCACCCTC	194	0,0000	0,0000	0,0011	0,0405
Lead 195	GCAATGTGTTCTGTCACGCATAGCAGTCC	195	0,0000	0,0000	0,0011	0,0639
Lead 196	GTCGGGAGATTCTCTGGACGGCCCACCTGT	196	0,0000	0,0000	0,0021	0,0405
Lead 197	CAGTCTGAGCAGATGTGGGTCGCCGCTTCT	197	0,0000	0,0000	0,0011	0,0396
Lead 198	CCACAGTACGCCCGCCGATACCGGGGTTG	198	0,0000	0,0000	0,0074	0,0630
Lead 199	GGACTGATGGACACCGTCGCGAGTCTGTCT	199	0,0000	0,0000	0,0011	0,0396
Lead 200	AAGCCTTCCGTGCTCTGGGTCGCCGGAGCC	200	0,0000	0,0000	0,0000	0,0387
Lead 201	AATTCGCGCGTATTCTGAGCCGCACCGAGT	201	0,0000	0,0000	0,0021	0,0567
Lead 202	CGACGTGTGCAGTAGCGGTCTGTTACTGGCC	202	0,0000	0,0000	0,0021	0,0387
Lead 203	GAGATTTCCTGGACTCGGGTACCAGTCACT	203	0,0000	0,0000	0,0042	0,0504
Lead 204	GAGTGTTTTCTCCGGCATTGGGTCGCCAGG	204	0,0000	0,0000	0,0011	0,0387
Lead 205	GAGTTGTTCCCGACCTCGAAGTCGGCTCCC	205	0,0000	0,0000	0,0021	0,0387
Lead 206	GCCCGTCCGTCCCTCGGTTCTGCCACCAAC	206	0,0000	0,0000	0,0000	0,0387
Lead 207	GGAGATTGGGTCGCCAAGTCCCCCGCTCTC	207	0,0000	0,0000	0,0000	0,0387
Lead 208	ACGCACATCGCTGTTCTGGTTGGGTCGCCA	208	0,0000	0,0000	0,0021	0,0378
Lead 209	ACGTACGGGTGGGATATGTCGATTGCCCCC	209	0,0000	0,0000	0,0011	0,0369
Lead 210	CTGACGAACCGTGCCGCTAGTCCGAGTGTC	210	0,0000	0,0000	0,0042	0,0369
Lead 211	GCATTTCCTCGGCCCCGATGGGGGTCAGTG	211	0,0000	0,0000	0,0021	0,0369
Lead 212	GCCACGTCCGATGAGCTCGACTCGCGTCCC	212	0,0000	0,0000	0,0011	0,0369
Lead 213	TGCGACCGTCTGTACGACCGCTACGAGTGC	213	0,0000	0,0000	0,0000	0,0369
Lead 214	AATCTTCTGGGCCGTCCCTCCGTACGTGT	214	0,0000	0,0000	0,0021	0,0360
Lead 215	AGGGATCGTCGGTTCCCGGCTGTCGGCCTA	215	0,0000	0,0000	0,0000	0,0360
Lead 216	CACCCCCTACGAATTTCCTACCGATGCCCC	216	0,0000	0,0000	0,0021	0,0360
Lead 217	GATTGACGCTAGTATGGGTCGCCAAGCTCC	217	0,0000	0,0000	0,0000	0,0351
Lead 218	AAGCCACTGCTCGCACTCTGGGTCGCCAGA	218	0,0000	0,0000	0,0000	0,0342
Lead 219	ACCGAGTCGCTCTTCTGGACGGCAACCAGT	219	0,0000	0,0000	0,0011	0,0342
Lead 220	ACTGTCCTGTGCTGGTTCTGCCGATGCTAC	220	0,0000	0,0000	0,0000	0,0342
Lead 221	ATGGGAGCTCTGGGTCGCCGGAGTGCCCTC	221	0,0000	0,0000	0,0000	0,0342
Lead 222	CAGTGACGCAGCACGACGTCCTCTCACCCG	222	0,0000	0,0000	0,0021	0,0342
Lead 223	CGGGATTGCTGAGCAGATGTGCGATCGAGT	223	0,0000	0,0000	0,0011	0,0342
Lead 224	GATCCTCTCGCTTCCGCGATCGCCCCACTG	224	0,0000	0,0000	0,0021	0,0342
Lead 225	GCAGCTAGAGTATGGGTCGCCACGCTCTAC	225	0,0000	0,0000	0,0011	0,0342
Lead 226	GGCGATACCCGATGCGTGGCTATTCGCCTG	226	0,0000	0,0000	0,0032	0,0342
Lead 227	ACTCGGGGGAAGCCTGCTCGTCGGTGCAGG	227	0,0000	0,0000	0,0000	0,0333
Lead 228	AGGCCATCGCCTTTCTGAGCCGGTGCGTGT	228	0,0000	0,0000	0,0011	0,0333
Lead 229	CATTCCTCTCGGTGGAGTTGGAGGTTATCA	229	0,0253	0,0293	0,0328	0,0171
Lead 230	ATGGGCCCATGTGGTCGCTCATGGGAGTCC	230	0,0000	0,0000	0,0021	0,0324
Lead 231	ATGTGGAGCCTCGTCCCGGGTGCCGGTCCC	231	0,0000	0,0000	0,0000	0,0324
Lead 232	CGAGATGTCGATTGAGGGCCCCCAGTAGTG	232	0,0000	0,0000	0,0032	0,0324
Lead 233	CGGGCCAACATTGCTCGTCGGTGCAGGGTT	233	0,0000	0,0000	0,0000	0,0819
Lead 234	GAGTTGGGTCGCCAGCTCACCAGGGTTTGC	234	0,0000	0,0000	0,0011	0,0324
Lead 235	GATGGAGGTCCTTGGGTCGCCACAGTAGCC	235	0,0000	0,0000	0,0042	0,0324
Lead 236	GCCCGTCCGCAACAGACTACGCGGGTCCCC	236	0,0000	0,0000	0,0032	0,0324
Lead 237	AATTTCCTGGATCCCTGATCACGACCCGAC	237	0,0000	0,0000	0,0000	0,0315
Lead 238	ACTCCTGTACCACCCTCTGGACGATGCTAC	238	0,0000	0,0000	0,0011	0,0315
Lead 239	GATAGAGCGCTAGTTCCTCGACCTACTCTG	239	0,0000	0,0000	0,0011	0,0621
Lead 240	GCATCGTTGTGACGTTGGGTCGCCACGTAC	240	0,0000	0,0000	0,0000	0,0315
Lead 241	GCTCGTCACCGGGCACTCCCCACGGTTCTC	241	0,0000	0,0000	0,0000	0,0315
Lead 242	GCTCGTCGCCGGAGCCCTCGCCGGCGTCCC	242	0,0000	0,0000	0,0000	0,0315
Lead 243	ACACGCGTTGGGTCGCCACCGCTCTGTTGC	243	0,0000	0,0000	0,0000	0,0306
Lead 244	ACCAAGGTGGCTGGTGCGCCCCGTCTCCCC	244	0,0000	0,0000	0,0063	0,0450
Lead 245	AGCATTTCTGGACCAGGGCGTCGCATTGAGT	245	0,0000	0,0000	0,0000	0,0306
Lead 246	CATCGACGTAGCGATCGACTACGCGGTCCT	246	0,0000	0,0000	0,0032	0,0306
Lead 247	CGACGACACCATTGGGTCGCCAAGGGGCCC	247	0,0000	0,0000	0,0011	0,0306
Lead 248	GAGTAGCTCCGTCCCCACCCAAGGGTCTCC	248	0,0000	0,0000	0,0011	0,0306
Lead 249	GCCCGACCGGTTGGCGGTCCCGCCTGTTCT	249	0,0000	0,0000	0,0000	0,0306
Lead 250	GGACCGGCAGAGATTCAGTTCCTACGCCGA	250	0,0000	0,0000	0,0000	0,0306
Lead 251	TAGTGGCGCGGTTGGGTCGCCAGCCGTCCC	251	0,0000	0,0000	0,0000	0,0306
Lead 252	AACGGATGCGGGATGTCTGCCCGTTCCGTG	252	0,0000	0,0000	0,0021	0,0423
Lead 253	ATGCTCTTGCCCATCTGGGTCGCCAGGTG	253	0,0000	0,0000	0,0011	0,0297
Lead 254	CATGGACTGCGGTATGGGTCGCCAGCTCGC	254	0,0011	0,0000	0,0021	0,0297
Lead 255	CGCAATTTCCTCGATGCCCCATCAGTGCCA	255	0,0000	0,0000	0,0021	0,0297
Lead 256	CTGAGCAGTTGGGTCGCCAGCGTATCCGTG	256	0,0000	0,0000	0,0000	0,0450
Lead 257	GCCCGTCAGCCCCAGGTCCCCTGGCTTCCC	257	0,0000	0,0000	0,0021	0,0297
Lead 258	AACCCCTTGCAGCGCCTGATTGGGTCGCCA	258	0,0000	0,0000	0,0021	0,0288
Lead 259	AGGCGAGCTTCTGAGCCGTGTACTCCGAGT	259	0,0000	0,0000	0,0032	0,0288
Lead 260	ATCCTCTCGTTCCTCCACGATGCCAGCGTG	260	0,0000	0,0000	0,0000	0,0288
Lead 261	ATGGAGTCTGGGTCGCCAGAGTCCACACCT	261	0,0000	0,0000	0,0000	0,0288
Lead 262	CAGGCGCAGGATATTGTCTGTCGATGCTCC	262	0,0000	0,0000	0,0011	0,0288
Lead 263	CATTTCCTGAGCCGACGCCACGCACCGAGT	263	0,0000	0,0000	0,0011	0,0288
Lead 264	CGTGTCGAGATTCTGAGCCGGATCTCGAGT	264	0,0000	0,0000	0,0021	0,0288
Lead 265	GAATTCGACCAGGCCGGAAGACCGTGTGCT	265	0,0000	0,0000	0,0042	0,0288
Lead 266	GACCTCGGCTGTTCTGCATCGATGCTACGT	266	0,0000	0,0000	0,0000	0,0288

Lead 267	GCTCCATTGCCCCCAGCTGTTCTTGACGCCA	267	0,0000	0,0000	0,0011	0,0288
Lead 268	TGCTGCGGACCCATTGCGTGCCGAGCTGCT	268	0,0000	0,0000	0,0000	0,0288
Lead 269	ACCCTAGGAAGCGCTCCTTCTGGGGGCCTA	269	0,0000	0,0000	0,0000	0,0279
Lead 270	ATGCGCGCTGATTCCTGACTTGGGTCGCCAC	270	0,0000	0,0000	0,0000	0,0279
Lead 271	CTCACTCGAGTATTCTGGACGGATCCCAGT	271	0,0000	0,0000	0,0000	0,0459
Lead 272	GACCTCCCACGTGCTGAGCAAGCGCTCCTT	272	0,0000	0,0000	0,0011	0,0279
Lead 273	GAGCGATTCGAGGCGTCGCTCCGAGTGTTC	273	0,0000	0,0000	0,0042	0,0279
Lead 274	GCTCGTCCCCGCTCTTCCAGCAAGGGTCCC	274	0,0000	0,0000	0,0032	0,0279
Lead 275	GACGTCCGCGAACGATGGCGCGCCCCCATT	275	0,0000	0,0000	0,0021	0,0270
Lead 276	GAGGACCTAGATGTACGTTGGGTCGCCACC	276	0,0000	0,0000	0,0000	0,0270
Lead 277	GCTGATGTCCTGGAACCGAGTTCATCGAC	277	0,0000	0,0000	0,0000	0,0270
Lead 278	GCTGCCTCGATGCTACGTGGGCGCCCGGTG	278	0,0000	0,0000	0,0011	0,0270
Lead 279	CACCAGCAGTGCAGTTGAGCCGCTACGAGT	279	0,0000	0,0000	0,0021	0,0261
Lead 280	CACTCCAGTGACCACGGGTCCTCTCACCCT	280	0,0000	0,0000	0,0011	0,0261
Lead 281	CCTATCCGCAGAGACTGTGGGTCGCCACAT	281	0,0000	0,0000	0,0000	0,0261
Lead 282	CCTCTCCCCAGTTCTGAGCCGTCGCGTGT	282	0,0000	0,0000	0,0000	0,0396
Lead 283	GACCCCGTGCTCCGTCATTATGGCCCCCAG	283	0,0000	0,0000	0,0032	0,0261
Lead 284	GACTCGTTGGGCCAGTCTTGCGACCTGTTC	284	0,0000	0,0000	0,0000	0,0261
Lead 285	GCCCGGAGAGATTCCTGAGCCGAACCGAGC	285	0,0000	0,0000	0,0011	0,0261
Lead 286	GCGTTTCTGGGCCGAGAATGCTCGTCGTGT	286	0,0000	0,0000	0,0000	0,0261
Lead 287	GTCGAATTCCTGAGCCGACTCTCATCGAGT	287	0,0000	0,0000	0,0032	0,0261
Lead 288	GTCGCTATGTCGATGCGCGCACCGCAACGG	288	0,0000	0,0000	0,0021	0,0261
Lead 289	TAGTGACCGACTTTCTGGACGGGCCCCAGT	289	0,0000	0,0011	0,0000	0,0261
Lead 290	AAGTTCCTGGATGCCGCACGGGATCACTC	290	0,0000	0,0000	0,0032	0,0252
Lead 291	AATCCCCGTTGCGACTTCGACCGATGCTCC	291	0,0011	0,0000	0,0000	0,0252
Lead 292	ACACTATTTCGGAGCCGGACCCCCCCGTGT	292	0,0000	0,0000	0,0032	0,0252
Lead 293	AGCAATTGTTACGTCCCAGACGCGCGAGTT	293	0,0000	0,0000	0,0032	0,0252
Lead 294	AGGCCATCTGGGTGTGCTCTGCATGGGGAT	294	0,0000	0,0000	0,0000	0,0252
Lead 295	CACCGAGGTTTTCTGTAGCCGCTCCGAGT	295	0,0000	0,0000	0,0000	0,0252
Lead 296	CAGCCGGTTGGGTCGCCAGTAGGCTTCCTA	296	0,0000	0,0000	0,0000	0,0252
Lead 297	CAGTAGTTTCTGCGACGAGTCATCTGTCCC	297	0,0000	0,0000	0,0011	0,0252
Lead 298	CGGGACGGGGCGGATTATGTCCCGGGTGCC	298	0,0000	0,0000	0,0000	0,0252
Lead 299	GAGATTGATGAGCCAGGCCTGCGTTGTGT	299	0,0000	0,0000	0,0011	0,0252
Lead 300	GAGTACTTGCGGAAGGCCCTGCCGCTGTTC	300	0,0000	0,0000	0,0053	0,0252
Lead 301	GAGTTCCCCGCGGGCAGGTTGTCTAGTCCCG	301	0,0000	0,0000	0,0000	0,0252
Lead 302	GAGTTGATTGGCGTCGACCGACCCTGTTCT	302	0,0000	0,0000	0,0011	0,0252
Lead 303	GTCTCGTCCGGACCACCTCGTACGGTCTAC	303	0,0000	0,0000	0,0011	0,0252
Lead 304	GTGCGAATGTCGCCGAGAGTGTCGTTCCG	304	0,0000	0,0000	0,0011	0,0252
Lead 305	GTTCCTCGAGAGAAGCTCTCCTCGAACCGC	305	0,0000	0,0000	0,0011	0,0252
Lead 306	ACACGACGCGGATATTCTGGACGATGCTAC	306	0,0000	0,0000	0,0011	0,0243
Lead 307	ACCTGTGGGGATTGTGAGCCGCAACCGAGT	307	0,0000	0,0000	0,0021	0,0243
Lead 308	ACGACGCTTGAGCCGCCACGAGTGTCATCC	308	0,0000	0,0000	0,0011	0,0243
Lead 309	CACGACAGCGGTTACTGACTTGGGTCGCCA	309	0,0000	0,0000	0,0032	0,0243
Lead 310	CGCAATCCGGAATACGACTTGGGTCGCCAG	310	0,0000	0,0000	0,0032	0,0243
Lead 311	GAGCTCGGCTGTCATGTTCTTGCCCCGTCGG	311	0,0000	0,0000	0,0011	0,0243
Lead 312	GAGGTCCGGGGTTTCTGTACCGGTTCCCTG	312	0,0000	0,0000	0,0000	0,0243
Lead 313	GCCCGGATTCCCCTGACTTGGGTCGCCACC	313	0,0000	0,0000	0,0000	0,0243
Lead 314	GCCCGTCCACGATCTCCTGCATCGTGTCCC	314	0,0000	0,0000	0,0000	0,0243
Lead 315	GGCTTGGGTCGCCACGCCACGCTGGTACGT	315	0,0000	0,0000	0,0011	0,0243
Lead 316	ACCTGAGGTCCGATGTCGATGGACTCTAGG	316	0,0000	0,0000	0,0011	0,0234
Lead 317	ACGGCCTTGCGGCCCCATGGAGAGCTATTC	317	0,0000	0,0000	0,0000	0,0234
Lead 318	AGCTGGTCGTCGGTCCAGTCGCATCTAGCA	318	0,0000	0,0000	0,0021	0,0405
Lead 319	CAATCTGGGTCGCCAGAGTGAGCTATCGTG	319	0,0000	0,0000	0,0011	0,0234
Lead 320	CCACGCGTCCGCATCTGGGTCGCCAGATGA	320	0,0000	0,0000	0,0011	0,0234
Lead 321	CCCCGAGGACCAGACTGTGGGTCGCCATCA	321	0,0000	0,0000	0,0021	0,0234
Lead 322	CTCCGCCAGTCTGGGTCGCCAGGACGTGCT	322	0,0000	0,0000	0,0011	0,0234
Lead 323	CTTGCGGAGCTGTTCTTGGACTGGCACCCG	323	0,0000	0,0000	0,0011	0,0234
Lead 324	GCTGTACATCTACGTCCCCAGGGGGGTCCC	324	0,0000	0,0000	0,0021	0,0234
Lead 325	TAGCGTGGTATTCCTCGAACGGCTCCCAGT	325	0,0011	0,0000	0,0021	0,0234
Lead 326	AATGTTGGCCCACGTTCTGTACGATGCTAC	326	0,0000	0,0000	0,0000	0,0225
Lead 327	AGCAGTGTATGGGTCGCCAGGCCTGCTCTG	327	0,0000	0,0000	0,0000	0,0378
Lead 328	AGGCCGCTCGCTTGTCCTTTGCGGAGTGTG	328	0,0000	0,0000	0,0021	0,0225
Lead 329	AGGCCTTGCGGAGCTCTTCTTGACCACCGA	329	0,0000	0,0000	0,0000	0,0225
Lead 330	AGGCGTTCGTAGCCAGTAGGCGCAGTGTTC	330	0,0000	0,0011	0,0042	0,0225
Lead 331	AGGTGCGGGGATCTGAGCCGTTACTCGAGT	331	0,0000	0,0000	0,0000	0,0225
Lead 332	ATCGGTTGGGTCGCCACCCGTCTCGCCTAT	332	0,0000	0,0000	0,0000	0,0225
Lead 333	ATTCGGTGCGTCCCGTCCGGATCCCGTCCA	333	0,0000	0,0000	0,0021	0,0225
Lead 334	CAATTCCGAGATTTCCGAGCCGTTCCGAGT	334	0,0000	0,0000	0,0000	0,0225
Lead 335	CAGCGGACCCAGCTTGGGTCGCCAGCCTGG	335	0,0000	0,0000	0,0011	0,0225
Lead 336	CATCGGTGGAGTTGGGTCGCCAAGTCACTG	336	0,0000	0,0000	0,0011	0,0225
Lead 337	CCAATGACGGAAGGCCGTCCTCTCATCGGT	337	0,0000	0,0000	0,0011	0,0225
Lead 338	CGGAGGACTACGATGCTACGTTGGGCTCCC	338	0,0000	0,0000	0,0042	0,0225
Lead 339	CTCGTTGGGTCGCCAGCGACGGGTCCCAGG	339	0,0000	0,0000	0,0000	0,0225
Lead 340	GACCCCTTTCTGTACGCCGAACACGTGTCT	340	0,0000	0,0000	0,0000	0,0225
Lead 341	GAGGTGCGCCCGTCGCCCTGGGTCGTCTAC	341	0,0000	0,0000	0,0011	0,0225
Lead 342	GCCATCCCGAGTCTTCTGTACGATGCTAC	342	0,0000	0,0000	0,0000	0,0225
Lead 343	GCCGGTCATGTTCTGGTCCCCCAACCGGTG	343	0,0000	0,0000	0,0042	0,0225
Lead 344	GCCGTCCAGGCTTTCTGAGCCGGTTCGAGT	344	0,0000	0,0000	0,0000	0,0225
Lead 345	TACTGTGGGTCGCCACACGAAGCGGACTCG	345	0,0000	0,0000	0,0011	0,0225
Lead 346	AGCCAACTCGCCTCTACTCTGGGTCGCCGGA	346	0,0000	0,0000	0,0011	0,0216
Lead 347	ATTGGGTCGCCAATGCCTCGGCCCCGTCAC	347	0,0000	0,0000	0,0000	0,0216
Lead 348	CAGCACTTCTGAGCCGGCCTCGTGGCGAGT	348	0,0000	0,0000	0,0011	0,0216
Lead 349	CGCCCAGTGTGGGTCGCCACACCCCGCGTG	349	0,0000	0,0000	0,0000	0,0324
Lead 350	GCCACGTCATGGAGCGGTCCTCCATGTCCC	350	0,0000	0,0000	0,0032	0,0216
Lead 351	GCCCGTCAGCAGCGTACGCTCGGCTGTCCC	351	0,0000	0,0000	0,0032	0,0216
Lead 352	GCGCAGTAGTTGGGTCGCCAGCGATGGCAG	352	0,0000	0,0000	0,0000	0,0216
Lead 353	GCTCGTTACTCGACCCAGTGGTCGAGTCCC	353	0,0000	0,0000	0,0032	0,0216
Lead 354	GCTCTGGGTCGCCACGAGTCGCATCGCAAG	354	0,0000	0,0011	0,0000	0,0216
Lead 355	AATAGCGATGCTGGGCGCCGGTCATGTTCT	355	0,0000	0,0000	0,0021	0,0207
Lead 356	AGAGCGTCTAGTTGGCCTCGCGCCTGTTCA	356	0,0000	0,0000	0,0011	0,0207
Lead 357	ATCACTCGCCCGACGACTCTCTGGATGCCG	357	0,0000	0,0000	0,0000	0,0207
Lead 358	CAGTGCGCCCGACCATATTCTTGACCTGTG	358	0,0000	0,0000	0,0000	0,0207
Lead 359	CCTAGTCTGGGTCGCCAGCGGGCCACCCTC	359	0,0000	0,0000	0,0000	0,0207
Lead 360	CGCAGCCATTTCCCGTCTGGACGATGCTAC	360	0,0000	0,0000	0,0000	0,0207
Lead 361	CGTTGGGTCGCCAGCGACATGCCCTTGCC	361	0,0000	0,0011	0,0000	0,0207
Lead 362	GAGAGCCGATACGAGTGTCTCGCGTTCGGT	362	0,0000	0,0000	0,0011	0,0207
Lead 363	GAGCTATTGGGTCCACGGAGTAGGCCGTTC	363	0,0000	0,0000	0,0000	0,0207
Lead 364	GCATAGCTGAGCCGAACTCCGTGTTCGAGT	364	0,0000	0,0000	0,0021	0,0207
Lead 365	GCCCCGCCCATATGTTTCTGTACGCGCTCC	365	0,0000	0,0000	0,0021	0,0207
Lead 366	GGATTACTGAGCCGGCCAGACGATCGTGT	366	0,0000	0,0000	0,0000	0,0207

Lead 367	GGTTGGGTCGCCACACCGTTCCCCATCGTG	367	0,0000	0,0000	0,0032	0,0207
Lead 368	GTGCCACGTCTGTCGATGCTACGTCGGAC	368	0,0000	0,0000	0,0000	0,0207
Lead 369	GTTGGGTCGCCAGCGCTGTCTCCACACCCG	369	0,0000	0,0000	0,0000	0,0207
Lead 370	AACCGGATTTGCAATCAGACGATCGGATTC	370	0,0165	0,0158	0,0201	0,0090
Lead 371	AGGTCGAGAGCACGTTGGGTCGCCAATGTG	371	0,0000	0,0000	0,0021	0,0315
Lead 372	CCATGCAGCTGACCGAACGCACTCAGTGTC	372	0,0000	0,0000	0,0000	0,0198
Lead 373	CCGGTCGGACAGTTGGGTCGCCAGCAACCG	373	0,0000	0,0000	0,0000	0,0198
Lead 374	CGAGTCTGCAGCCGATTGACCGGAAGTCGA	374	0,0000	0,0000	0,0021	0,0198
Lead 375	GAACCTGTTATCGTGGACCGCAATGCGAGT	375	0,0000	0,0000	0,0000	0,0198
Lead 376	GACACGTCATGTTCTTGGCCGCCGAGACCG	376	0,0000	0,0000	0,0011	0,0198
Lead 377	GCCAGTCCCCAGCGTGTCGTTGGGGTCCTC	377	0,0000	0,0000	0,0021	0,0198
Lead 378	GGAGTTTGTGGACCGCATCTCCACACGAGT	378	0,0000	0,0000	0,0000	0,0198
Lead 379	GTCAGGAGCGGAACCCAGTGTCCTGTAGCT	379	0,0000	0,0000	0,0011	0,0198
Lead 380	TCTCGATGCTACGTGAGCATGGCGGGGGT	380	0,0000	0,0000	0,0000	0,0198
Lead 381	AACACGTTGGGTCGCCAGCGCGTGTCATCT	381	0,0000	0,0000	0,0000	0,0189
Lead 382	AACCTCCTGCTATTTCGTACGGCTCCCAGT	382	0,0000	0,0000	0,0000	0,0189
Lead 383	AATCTTCTGAGCCGCTTGTGGCAGTCGAGT	383	0,0000	0,0000	0,0021	0,0189
Lead 384	AATTTGTGAGCCAGGGGTATTCCGCCGAGT	384	0,0000	0,0000	0,0011	0,0189
Lead 385	ACCACGGTGGATCCCGTAGCGCTCCTTCGG	385	0,0000	0,0000	0,0032	0,0189
Lead 386	ACGAGTGTACCAGCCCAGCAGATGGTAGCC	386	0,0000	0,0000	0,0000	0,0189
Lead 387	ACGTGCGTCCCTCTCTGAGCAGATGCTACT	387	0,0000	0,0000	0,0011	0,0189
Lead 388	ATGGGTAGTGTGGTCGCTCACGCACCCCTC	388	0,0000	0,0000	0,0011	0,0189
Lead 389	CCGCCACGATTGCCATACGTGGTAGTCGGC	389	0,0000	0,0000	0,0000	0,0189
Lead 390	CTCCCACGCGCCACGTGCTTGTCGATGCAC	390	0,0000	0,0000	0,0032	0,0189
Lead 391	CTTGGTTGCTTTATCCATGCTTCGGCATTA	391	0,0099	0,0180	0,0106	0,0189
Lead 392	GAACCTCATCGACGGATCTGGGTAGGCACCA	392	0,0000	0,0000	0,0021	0,0189
Lead 393	GAAGGATTGGGTCGCCAGTCCCTCTCTGTG	393	0,0000	0,0000	0,0000	0,0189
Lead 394	GAAGTTACTGGGCCGGTGTCCTCCTCGAGT	394	0,0000	0,0000	0,0000	0,0189
Lead 395	GCCTCGTACCAAGTCCGCCACAGGTTCCCC	395	0,0000	0,0000	0,0021	0,0189
Lead 396	GCTAGTCCCGGCCTCTCGTACGCCGGTCCC	396	0,0000	0,0000	0,0000	0,0189
Lead 397	GGTATGGGCCGCTTGGGTCGCCATGCGGT	397	0,0000	0,0000	0,0011	0,0189
Lead 398	GTAGTTTCTGAGCCCATGTCGGTGT	398	0,0000	0,0000	0,0000	0,0189
Lead 399	AAGTTCTGAGCCGCACAACGTGACTCGTGT	399	0,0000	0,0000	0,0000	0,0180
Lead 400	AATGTCATCTCACTAGGCGCGTGGAGTAGG	400	0,0000	0,0000	0,0000	0,0180
Lead 401	ACCGAGGCCACATGTCGATTGGTGTCGGTG	401	0,0000	0,0000	0,0032	0,0180
Lead 402	AGCAGCGGATTGTCGATTCCGTGCTCACTT	402	0,0000	0,0000	0,0000	0,0180
Lead 403	CATGCCTCCACTGGACTCTTGGTCGCCAGA	403	0,0000	0,0000	0,0000	0,0180
Lead 404	CATGTCCTCTCACCTCGACGTGTCCCATCC	404	0,0000	0,0000	0,0000	0,0180
Lead 405	CTCTCCGGCTCTGGGTCGCCACGAGCCCTA	405	0,0000	0,0000	0,0000	0,0180
Lead 406	GACCATCCATCGTCGGTTGGAGGCGCGGTG	406	0,0000	0,0000	0,0000	0,0180
Lead 407	GCAGGTCATCTCACCCCACGACACGTGACC	407	0,0000	0,0000	0,0011	0,0180
Lead 408	GCCCCGTCAGTCCCACCAACGGACGTCCTC	408	0,0000	0,0000	0,0032	0,0180
Lead 409	GCGGAGTTGGGTCGCCTACGCAGCGCAGTG	409	0,0000	0,0000	0,0042	0,0288
Lead 410	GGATACGATAGCAGTTGGAGCCGCCCCCGT	410	0,0000	0,0000	0,0000	0,0180
Lead 411	GGATTGGCCTCGTCGGTGGCAACCTGCGTG	411	0,0000	0,0000	0,0011	0,0180
Lead 412	GGCCTTCTCGTCCGAACCGCTCCGAGTGTC	412	0,0000	0,0000	0,0000	0,0180
Lead 413	GGGTCGGTTGGGTCGCCAAGCTCTCCGGTG	413	0,0000	0,0000	0,0021	0,0180
Lead 414	TAGCACCAGGTTGGGTCGCCAGCCTCGATC	414	0,0000	0,0000	0,0000	0,0180
Lead 415	TGTGGAGCCATGTTGCGTGTCCCAGCGCTG	415	0,0000	0,0000	0,0000	0,0180
Lead 416	AATACCGCCTACGGGGCGCCCGTCATGTTC	416	0,0000	0,0000	0,0011	0,0171
Lead 417	ACTTGGGTCGCCAGGGGCTAGGTTGTCTGC	417	0,0000	0,0011	0,0021	0,0171
Lead 418	AGATGTGCCTTGTTCTGAGCGGCCACCAGT	418	0,0000	0,0000	0,0000	0,0171
Lead 419	AGGCCGCCTCTCCTCGTCGGTGGAGAGACG	419	0,0000	0,0000	0,0000	0,0171
Lead 420	AGTATGGGTCGCCATAGTGCCCCCCCACCA	420	0,0000	0,0000	0,0011	0,0171
Lead 421	CACGGCGGATGTGGTCGCTCAGCCTCCGTG	421	0,0000	0,0000	0,0000	0,0171
Lead 422	CACTCGCGTCTGGGTCGCCAGACCGTTCAT	422	0,0000	0,0000	0,0000	0,0171
Lead 423	CAGCGCACACGATGCTACGTTGCCCCGGACT	423	0,0000	0,0000	0,0000	0,0171
Lead 424	CCGGCTCTGCGGTTTCGTACGGTTCCCAGT	424	0,0000	0,0000	0,0000	0,0171
Lead 425	CTCCTGCTAGGCCCTGATGTGGGTCGCCAC	425	0,0000	0,0000	0,0000	0,0171
Lead 426	GACCCGTCTCGTGCACCGCGCGAGTTCCCC	426	0,0000	0,0000	0,0011	0,0171
Lead 427	GAGATCATTGCCCACCTACGTAGGGTTCGC	427	0,0000	0,0000	0,0000	0,0171
Lead 428	GCCACGTCATGTTCTGCGGGTACGGGCCCT	428	0,0000	0,0000	0,0000	0,0171
Lead 429	GCCCGACCCATGTTCTTGACGCCCGCCTAT	429	0,0000	0,0000	0,0011	0,0171
Lead 430	GGAATCGCTTGGCTAGCGAGCATCAGTGTA	430	0,0000	0,0000	0,0011	0,0171
Lead 431	GGCCCCCCGGCATTGCCCCCAGGCTATTCT	431	0,0000	0,0000	0,0011	0,0171
Lead 432	AATCGAGGACTCTCGGGTCGCCAGAGGCC	432	0,0000	0,0000	0,0021	0,0162
Lead 433	AATGAGCGCCCGCCTCGCTCCTCTCATCTT	433	0,0000	0,0000	0,0000	0,0162
Lead 434	ACGCAGGTGATGTAGTCGATTGGGTCGCCA	434	0,0000	0,0000	0,0000	0,0162
Lead 435	AGGTTGTAGTGCTGCGCCAAGGTGCGGCTA	435	0,0000	0,0000	0,0000	0,0162
Lead 436	ATCTGACCCCGGTTGGGTCGCCAGACGGCT	436	0,0000	0,0000	0,0032	0,0162
Lead 437	ATGATCTCGGACGAGTGTCCCATTGGAGCC	437	0,0000	0,0000	0,0032	0,0162
Lead 438	ATGCCGTGTGGGTCGCCACAGGCCTACGTG	438	0,0000	0,0000	0,0000	0,0162
Lead 439	CAGGCTAGTCGTCGGTCTAGGCCAATTGA	439	0,0000	0,0011	0,0000	0,0162
Lead 440	CCCCGCCACGTGACCTTGCGGAGCTGTTCT	440	0,0000	0,0000	0,0000	0,0162
Lead 441	CCCGACGTGGTTTGTCGATCCACAGGACGG	441	0,0000	0,0000	0,0000	0,0162
Lead 442	CCGTCTCTGGGTCGCCAGAGTGGCTCGCTG	442	0,0000	0,0000	0,0000	0,0162
Lead 443	CGCCAGTAGCCCAGCGTATGTTCTTGACCT	443	0,0000	0,0000	0,0000	0,0162
Lead 444	GAACCATTTATGAGCCGCTCTAGCTCGAGT	444	0,0000	0,0000	0,0011	0,0162
Lead 445	GACTCGTTAGTGGCCAACCGCCCACTCCCC	445	0,0000	0,0000	0,0032	0,0279
Lead 446	GAGGAATTCATGGATCGGGCCCCGATCACT	446	0,0000	0,0000	0,0011	0,0162
Lead 447	GAGTTTCTCGGGCGCCATCGATAGCGGATA	447	0,0000	0,0011	0,0011	0,0162
Lead 448	GCACCGCATTTCTCACTTGGGTCGCCCAGT	448	0,0000	0,0000	0,0000	0,0162
Lead 449	GCCCGTCCTTCCCATGGGCGGAAGGTCTCC	449	0,0000	0,0000	0,0032	0,0162
Lead 450	GGCGAGATTTCCGTTTGAGCCAGCCTGAGT	450	0,0000	0,0000	0,0000	0,0162
Lead 451	GGGGCGATTGGGTCGCCAATCTCCACGTCC	451	0,0000	0,0000	0,0000	0,0162
Lead 452	GGGGTCCTGAGCCGACAATCTCATGTCGAGT	452	0,0000	0,0000	0,0032	0,0162
Lead 453	TGCTGCCATTGGGTCGCCGAGGGCATCGCT	453	0,0000	0,0000	0,0000	0,0162
Lead 454	AACCAGACTCTTCGGGCTTGGGTCGCCAAG	454	0,0000	0,0000	0,0000	0,0153
Lead 455	ACTACGCGGGATTGGGTCGCCACATCCAGT	455	0,0000	0,0000	0,0000	0,0153
Lead 456	AGTACTGCTCCCCGTCTCCCCCAGAGTCCC	456	0,0000	0,0000	0,0011	0,0153
Lead 457	AGTATTTGTGAGCCGTACCCCCACTCGAGT	457	0,0000	0,0011	0,0011	0,0153
Lead 458	ATCTGTAGTCCTTGGGTCGCCAGGGAGGTG	458	0,0000	0,0000	0,0000	0,0153
Lead 459	CAATTTCCTGGACCACGGGTATCGACGAAC	459	0,0000	0,0000	0,0000	0,0153
Lead 460	CCCCCGTCCGATGTCGATGGACCTGCAGTG	460	0,0000	0,0000	0,0011	0,0153
Lead 461	CCCTGCGATTTAGCTGGACCAGCCATGAGT	461	0,0000	0,0000	0,0000	0,0153
Lead 462	CCGCACAGGTTGGGTCGCCAGGCCGAGTCG	462	0,0000	0,0000	0,0000	0,0153
Lead 463	CCTCATCGAACGCCACCCGATTCCTCGAGG	463	0,0000	0,0000	0,0011	0,0153
Lead 464	CGAGGTGTTGGTCGCTACACCCATCCGGCC	464	0,0000	0,0000	0,0000	0,0153
Lead 465	CTCTTGCGCAGTTCCTGGACGGCCCCCAGT	465	0,0000	0,0000	0,0000	0,0153

Lead 466	GACTCCTCTCTATGGCAATGAGCGCACCGG	466	0,0000	0,0000	0,0011	0,0153
Lead 467	GAGCCAGATTCTACATGGTGGTCGCTCCAT	467	0,0000	0,0000	0,0011	0,0153
Lead 468	GAGCTCCGTCCCTGGTGGTTACCAGGTCCC	468	0,0000	0,0000	0,0000	0,0153
Lead 469	GCTGTGGGTCGCCACAGCCCGCTGCTCCCC	469	0,0000	0,0000	0,0000	0,0153
Lead 470	GGATTCCCGTCCCCCCGTGGTTCTGGCCTC	470	0,0000	0,0000	0,0021	0,0153
Lead 471	GGCCTAGCACAGGCTCCGTTCCTTGTGCCC	471	0,0000	0,0000	0,0000	0,0153
Lead 472	GTAGCGGTTATGCCCGGATTGGGTCGCCAA	472	0,0000	0,0000	0,0000	0,0153
Lead 473	GTATTGAGCGGAGTTCCTCGACGCCCACC	473	0,0000	0,0000	0,0011	0,0153
Lead 474	GTCCGGGCGAACGCAGGTTGTCTAGTCGGA	474	0,0000	0,0000	0,0000	0,0153
Lead 475	GTTGGGTCGCCAGCGCTACGCGCCGACGTG	475	0,0000	0,0000	0,0000	0,0153
Lead 476	AACTGCACGTTCGACCTTGGGTCGCCATAG	476	0,0000	0,0000	0,0000	0,0144
Lead 477	ACCCATTGGGATTCTGGGCCGGTTCCGAGT	477	0,0000	0,0000	0,0000	0,0252
Lead 478	AGACGATTCCTGAGCCGTACTCTGCGAGT	478	0,0000	0,0000	0,0000	0,0144
Lead 479	AGGAGCACATACGTGCGCGACGGGTGCCTG	479	0,0000	0,0000	0,0021	0,0144
Lead 480	AGTCGAGTCTGGGTCGCCAGGATTCGTCTC	480	0,0000	0,0000	0,0011	0,0144
Lead 481	AGTGAGTCGCGCAACGCCATTGGGTCGCCA	481	0,0000	0,0000	0,0000	0,0144
Lead 482	ATGGATCCTCTGACAGCCGACGACGAGTGT	482	0,0000	0,0000	0,0011	0,0144
Lead 483	ATTCGGGCTGGGTCGTCGGTCCCTGCCGGT	483	0,0000	0,0000	0,0000	0,0144
Lead 484	CAGGTCCTCTCGTTCTCACGACCGGTGTG	484	0,0000	0,0000	0,0000	0,0144
Lead 485	CATGTAGGTCGCTGCAGGGCGCCGACGAGG	485	0,0000	0,0000	0,0021	0,0144
Lead 486	CCATTCGGACAGGGTTGGGTCGCCAGCGCA	486	0,0000	0,0000	0,0000	0,0144
Lead 487	CCGAGTGGGCGCCCGTCCATGTTCTGGCTT	487	0,0000	0,0000	0,0000	0,0144
Lead 488	CCGCACCTTTCCTTCTGAGCCGCTGCGAGT	488	0,0000	0,0000	0,0000	0,0144
Lead 489	CTGCAGTCGATTGTCGATTCGCTGCCCCGC	489	0,0000	0,0000	0,0000	0,0144
Lead 490	CTGCTCCTTTCTGGGCCGACCCTGTCGAGT	490	0,0000	0,0000	0,0000	0,0144
Lead 491	GACTGCCGTTCCTTGTCGATGGAGCGAGAG	491	0,0000	0,0000	0,0000	0,0252
Lead 492	GACTTTGCCGACGCTGTTCTTGACGCTCAC	492	0,0000	0,0000	0,0000	0,0144
Lead 493	GAGGATTCTGAGCCATTGGCAGAGATGAGT	493	0,0000	0,0000	0,0000	0,0144
Lead 494	GATAGGCGCACTGTGGTCGCTCAGAACGCC	494	0,0000	0,0000	0,0000	0,0144
Lead 495	GATCTCGGCCGTGCCAATGTCGATTGCCGT	495	0,0000	0,0000	0,0000	0,0144
Lead 496	GATCTGGGTCGCCGGATCGCGGTTCCCTCA	496	0,0000	0,0000	0,0000	0,0144
Lead 497	GATGGCTTGCCCGCTCTTGTTCCTCCGCCC	497	0,0000	0,0000	0,0000	0,0144
Lead 498	GCATTCCCTCGTCGGTGGGGATGCTCCGG	498	0,0000	0,0000	0,0000	0,0144
Lead 499	GCCAGTCCGCGTGGGCACTACGCCGGTCTC	499	0,0000	0,0000	0,0021	0,0144
Lead 500	GCCCGTAGTGCCGGCCTCTAGGCACTTCTC	500	0,0000	0,0000	0,0011	0,0144
Lead 501	GCGGACAATACTGGTGCGCCCCGTCTCCCC	501	0,0000	0,0000	0,0011	0,0144
Lead 502	GGCTGTGGACGAGTATCAGTGCTACCGCCC	502	0,0000	0,0000	0,0032	0,0144
Lead 503	GGGGATTGGGTCGCCAACTCCACATCGTCG	503	0,0000	0,0000	0,0011	0,0144
Lead 504	GGGTATGGGTCGCCAGGCTCGGTGCATGT	504	0,0000	0,0000	0,0011	0,0144
Lead 505	GTACTGGCTCTGGTGGTCGCTCCAGAGGTG	505	0,0000	0,0000	0,0000	0,0144
Lead 506	TACTCCTCATGACTGATCTGGGTCGCCACG	506	0,0000	0,0000	0,0000	0,0360
Lead 507	TAGCCATGACTCGTCGGTGTCGGGGCGGAT	507	0,0000	0,0000	0,0000	0,0144
Lead 508	TATTCTGGGTCGCCACGGATAGCACCAGG	508	0,0000	0,0000	0,0000	0,0144
Lead 509	AACTCGTGCTCCCCGTCCGTTCCGGTCCCC	509	0,0000	0,0000	0,0032	0,0135
Lead 510	AATGCCAGCCGATGCGAGTGTGGCAGCTAT	510	0,0000	0,0000	0,0000	0,0135
Lead 511	ACACCTCCCTGAGCTTTCTGGACGCGCTCC	511	0,0000	0,0000	0,0000	0,0135
Lead 512	ACACCTGGTCTGGGTCGCCAGAACTAGCGT	512	0,0000	0,0000	0,0000	0,0135
Lead 513	ACATTGGGTCGCCAGGGTCTGTCGGTACGTG	513	0,0000	0,0000	0,0000	0,0135
Lead 514	ACCCCAGGGGATCGTCGGTTCCCCTCTACT	514	0,0000	0,0000	0,0000	0,0135
Lead 515	AGATCCCTGAGCAACCGTATCCGAGTGTG	515	0,0000	0,0000	0,0021	0,0135
Lead 516	ATCCAGATGGGATTTCTCGCCGGTTCCCTG	516	0,0000	0,0000	0,0011	0,0135
Lead 517	ATTTCTGAGCGAGCCCTGTCGGTGCACTGT	517	0,0000	0,0000	0,0000	0,0135
Lead 518	CAATGCGACGTTTCTTGAGCGGGCCCCTGT	518	0,0000	0,0000	0,0021	0,0135
Lead 519	CAGACATGCTTGCGGCTTGCGCAGCTGTTC	519	0,0000	0,0000	0,0011	0,0135
Lead 520	CAGGACCTGGTTGGGTCGCCACACTTGTCC	520	0,0000	0,0000	0,0000	0,0135
Lead 521	CATCCGATTGGGTCGCCACATCTCGGCACT	521	0,0000	0,0000	0,0011	0,0135
Lead 522	CATGTGGACGTTTCGTGGACCCTCCGGAGT	522	0,0000	0,0000	0,0000	0,0135
Lead 523	CCATGCTGGGTCGCCACATGGGGATTCTCC	523	0,0000	0,0000	0,0000	0,0135
Lead 524	CCCAAGTCCCTGGTGTGGGTCGCCGCACCG	524	0,0000	0,0000	0,0032	0,0135
Lead 525	CGTAGCAGACGACTACGCAGGTGCACCCTT	525	0,0000	0,0000	0,0021	0,0135
Lead 526	GAACCCTGGCCTAGCTTGTCGATGCTGGCC	526	0,0000	0,0000	0,0011	0,0135
Lead 527	GACCTGGTCGCGAAGACTTGGGTCGCCATA	527	0,0000	0,0000	0,0000	0,0135
Lead 528	GCACGTACGGGCTGGCCCAGCCCGTTCCCC	528	0,0000	0,0000	0,0000	0,0135
Lead 529	GCATTTCTGAGCGGTCCTACTGGGTCCAGT	529	0,0000	0,0000	0,0000	0,0135
Lead 530	GCCGCTCCTTGTCGATGGAGCATAGCCACA	530	0,0000	0,0000	0,0000	0,0135
Lead 531	GCCGTGGGCCACATTTTCTACGCGCTAGGA	531	0,0000	0,0000	0,0000	0,0135
Lead 532	GCGCCAATGGACAGTAGTCGTGTCTCCGTG	532	0,0000	0,0000	0,0021	0,0135
Lead 533	GCGCCGGTGATGGGTCGCCATACCGCGCCC	533	0,0000	0,0000	0,0000	0,0135
Lead 534	GCTATCTCTTAGCCCCCGCTGGAGCTCCCC	534	0,0000	0,0000	0,0021	0,0135
Lead 535	GCTCGTCCCGATGAGGGTATTCTGGCGTCC	535	0,0000	0,0000	0,0032	0,0135
Lead 536	GGACAGTTGCTCGTCGGTGCACCAGTCCAG	536	0,0000	0,0000	0,0000	0,0135
Lead 537	GGGCTACGGCTGGATTGGGTCGCCAATCCC	537	0,0000	0,0000	0,0032	0,0135
Lead 538	GTTTCGGAGCCGCGGGGTCTTCCCTCGAGT	538	0,0000	0,0000	0,0000	0,0135
Lead 539	TGCTGGGTCGCCAGAGCGCAACGTCCATTT	539	0,0000	0,0000	0,0011	0,0135
Lead 540	AACTGTGACGCGCTTGGGTCGCCAGCAATA	540	0,0000	0,0000	0,0000	0,0126
Lead 541	ACCTGGAACTTTCTCGAACGGTCCGCCTGT	541	0,0000	0,0000	0,0000	0,0126
Lead 542	ACTCGGGCCACATCGATTACGACCGTACCC	542	0,0000	0,0000	0,0042	0,0126
Lead 543	AGAGGCCAAGGCTGACGTGTGGTCGCTCAC	543	0,0000	0,0000	0,0000	0,0126
Lead 544	AGATTGGGTCGCCAATCATAGCCCCCGCGTG	544	0,0000	0,0000	0,0000	0,0126
Lead 545	ATATTCTGGGCCGGACTTCCAGTCACGAGT	545	0,0000	0,0000	0,0000	0,0126
Lead 546	ATCGGAGCCGACATCGAGTGTCCGAGCTTC	546	0,0000	0,0000	0,0000	0,0126
Lead 547	ATCTGTGCGCCCGTCATGTTCTCGGCCTCG	547	0,0000	0,0000	0,0021	0,0126
Lead 548	CAACCCATTCATTTCCGAGCCGCACCGAGT	548	0,0000	0,0000	0,0000	0,0126
Lead 549	CAGCATACCGAAGTCTGGACGGTTCCCAGT	549	0,0000	0,0000	0,0011	0,0126
Lead 550	CAGCGCGTGTCCTTTGCGGAGTGCTTAGTG	550	0,0000	0,0000	0,0000	0,0126
Lead 551	CATCGACCACAGTGGCGCCCCGTCATGTTC	551	0,0000	0,0000	0,0021	0,0126
Lead 552	CCGAAGGTGGACGTGCGCCAGTCATGTTCT	552	0,0000	0,0000	0,0000	0,0126
Lead 553	CGATCCTCTCGTGTGCACGATCGCCATCCT	553	0,0000	0,0000	0,0000	0,0126
Lead 554	CGGGTAGGAGTTCTGGGCCGTGCAACGAGT	554	0,0000	0,0000	0,0000	0,0126
Lead 555	GACGCCGTATTTCCCTGCACATAGGGATCG	555	0,0000	0,0000	0,0021	0,0126
Lead 556	GACGTCGTTTCCGCCTGCTCGTCGGTGCA	556	0,0000	0,0000	0,0000	0,0126
Lead 557	GAGAGATTTTGCGACCGTGCCACGAGTCTC	557	0,0000	0,0000	0,0000	0,0126
Lead 558	GAGGTCATCTCGTCCTCTAGCGACCTCGGC	558	0,0000	0,0000	0,0021	0,0126
Lead 559	GCAGCCTCTCGCACTTTCTACGCGCAGGA	559	0,0000	0,0000	0,0011	0,0126
Lead 560	GCCAGGTTGGGTCGCCAGCCTGATGGCCAT	560	0,0000	0,0000	0,0000	0,0126
Lead 561	GCCTCGTTACGCTGCCACACGCAGAGTCCC	561	0,0000	0,0000	0,0021	0,0126
Lead 562	GCGGAGGCTCTTGTCCTTCCGCCCACGGTG	562	0,0000	0,0000	0,0000	0,0126
Lead 563	GGACAGGGCCCAGTGGTTCATCGACGGCAC	563	0,0000	0,0000	0,0000	0,0126
Lead 564	GGGGCATTGTGCAGCTTGGGTCGCCAGGTT	564	0,0000	0,0000	0,0011	0,0126
Lead 565	GGGGGGCCCGGCTGGTTGTCGATCCAGCAC	565	0,0000	0,0000	0,0021	0,0126
Lead 566	GGGTGCCAATCCTTCGGAGCCGTTCCGAGT	566	0,0000	0,0000	0,0000	0,0126

Lead 567	GGTAGCCCAGGGAGCATTGTCGATTGCCCC	567	0,0000	0,0000	0,0000	0,0126
Lead 568	GTAGTCTGCATCCAGCCGCCCGCGAGTGTG	568	0,0000	0,0000	0,0011	0,0126
Lead 569	GTCCCGTCCGCGGGTTATCCCCCGCGTCCA	569	0,0000	0,0000	0,0011	0,0126
Lead 570	GTCGTAGGCCCGAGTCCGATGTCGATGGCGA	570	0,0000	0,0000	0,0011	0,0126
Lead 571	TACCGTGGCCATTACTGAGCCGTACCGTGT	571	0,0000	0,0000	0,0000	0,0126
Lead 572	AATCGGGACGCGTAGTCTGGGTCGCCACTG	572	0,0000	0,0000	0,0000	0,0117
Lead 573	AATGTCGATTGCATGGAGGCGGCGGCAGTG	573	0,0000	0,0000	0,0000	0,0117
Lead 574	ACCACTCTTTCTGGACTTGGGTCGCCACCA	574	0,0000	0,0000	0,0000	0,0117
Lead 575	ACCCCTGCGGATGTCGATCGAGGCAAGCAT	575	0,0000	0,0000	0,0000	0,0117
Lead 576	ACCTCTGCATCGATTGGGTCGCCAGGCGTG	576	0,0000	0,0000	0,0000	0,0117
Lead 577	ACGAACGTAGTTCCTCGACGCGGCCCCCAC	577	0,0000	0,0000	0,0032	0,0117
Lead 578	ACGATGCCGAATGTTCTGAGCCAGTTGCGT	578	0,0000	0,0000	0,0000	0,0117
Lead 579	AGAGTGTGGAAGCCCCGTCCATGTTCTGCC	579	0,0000	0,0000	0,0000	0,0117
Lead 580	ATCGGCTAGCGCTCCTAGTCGGGCCCTCTC	580	0,0000	0,0000	0,0000	0,0117
Lead 581	ATGGACACCCCATTGTGGCTGATCCAGGCC	581	0,0000	0,0000	0,0000	0,0117
Lead 582	CACCCGGGTGGATTGTCGATTCCACGCCGC	582	0,0000	0,0000	0,0011	0,0117
Lead 583	CAGCGGTGTTACCTGAGTTGGGTCGCCAAT	583	0,0000	0,0000	0,0011	0,0117
Lead 584	CATCCTCCCTCTGCCACCCGTTCCGAGGCT	584	0,0000	0,0000	0,0011	0,0117
Lead 585	CGCGAACCCGTTGTCGACTGTGGTCGCTCA	585	0,0000	0,0000	0,0000	0,0117
Lead 586	GAGCATGCGTGGTTCATCGACGCTGTCGGC	586	0,0000	0,0000	0,0011	0,0117
Lead 587	GAGCCAAGTGGCTCCTCTCGCTTCCTCGTG	587	0,0000	0,0000	0,0000	0,0117
Lead 588	GAGGCCCGCCCTTTGGCACCATGCCTGTTC	588	0,0000	0,0000	0,0000	0,0117
Lead 589	GAGTATTCGTGTCAGGTTGGGTCGCCACCC	589	0,0000	0,0000	0,0000	0,0117
Lead 590	GATCGTTCTGAGCCGGGCTCGCATTCGTGT	590	0,0000	0,0000	0,0000	0,0117
Lead 591	GATGTCTGGTCGGCCGACTTTCTGGTCC	591	0,0000	0,0000	0,0000	0,0117
Lead 592	GCCAGTCCGATCGCCCAGTGACCGGTCTCC	592	0,0000	0,0000	0,0011	0,0117
Lead 593	GCCCCGTTACTCTCCGTCGCCGGAAGTCCC	593	0,0000	0,0000	0,0011	0,0117
Lead 594	GCCGCGTAGCATTTCTCAGCCGTTCCGAGT	594	0,0000	0,0000	0,0000	0,0117
Lead 595	GGAATTTCCTGGAGCGCCCGATCACTCGTC	595	0,0000	0,0000	0,0000	0,0117
Lead 596	GGAGTGTGAGTTCTGAGCGGACTCCCCAGT	596	0,0000	0,0000	0,0011	0,0117
Lead 597	GGCCGCCACTTTTCTGAGCCGATTGCGAGC	597	0,0000	0,0000	0,0000	0,0117
Lead 598	GGCCTGGTCGTCGGTCCAGCAATTCCCTT	598	0,0000	0,0000	0,0021	0,0117
Lead 599	GGGTCCTCTCGTCCTCCACGACCCAAGCAC	599	0,0000	0,0000	0,0032	0,0117
Lead 600	GTAGCCTCTCCCCAGCGCCCGTCATGTTC	600	0,0000	0,0000	0,0032	0,0117
Lead 601	GTCCTAGTTGGCCGGTACCTCCGCCTGTTC	601	0,0000	0,0000	0,0021	0,0117
Lead 602	GTCGCGGAGTTATCGTGCCACCAGTGAGC	602	0,0000	0,0000	0,0000	0,0117
Lead 603	TAGCTAGAGCCGCACCCGAGTGTCTTGCTA	603	0,0000	0,0000	0,0000	0,0117
Lead 604	TATGGGTCGCCATAGCTAGCGTATCCCTGC	604	0,0000	0,0000	0,0000	0,0117
Lead 605	TTGCGGACTTCTGCGCCGAGCAATCTGGCC	605	0,0000	0,0000	0,0011	0,0117
Lead 606	TACGTCGGTTCCTTTATCCATGGTTTCGTA	606	0,0044	0,0113	0,0085	0,0099
Lead 607	TCTCGGTGGAGTCGGAGGTATCTTGAATTG	607	0,0044	0,0113	0,0085	0,0045
Lead 608	AAGCTTGGGTCGCCCAGCGCGCGCACATTT	608	0,0000	0,0000	0,0032	0,0108
Lead 609	AATGGAGCCGTTCTGCTATGGGTCGCCAGG	609	0,0000	0,0000	0,0011	0,0108
Lead 610	ACACGGTCTTGGCAGCCCGCTCCGTGGTCT	610	0,0000	0,0000	0,0011	0,0108
Lead 611	ACAGGGGAGTTGGGTCGCCACGGCTCCCTG	611	0,0000	0,0000	0,0011	0,0108
Lead 612	AGACAGCGCCATTGGGTCGCCGATGAGACT	612	0,0000	0,0000	0,0032	0,0108
Lead 613	AGGCGACGATCTTCCTGAGCCACGTTGAGT	613	0,0000	0,0000	0,0011	0,0108
Lead 614	AGTCTGGGCAGACCGGACCGTCCGAGTGCT	614	0,0000	0,0000	0,0032	0,0108
Lead 615	AGTTGGGTCGCCAGCAGCCTACTGGACGGA	615	0,0000	0,0000	0,0011	0,0108
Lead 616	ATCACCTGCGCAGTCCTCGTCGGTGGAGTC	616	0,0000	0,0011	0,0000	0,0108
Lead 617	CACGGGAGTCCACCCCTTTGGCCATCAGTG	617	0,0000	0,0000	0,0011	0,0108
Lead 618	CATGTGTGACTATTCTGGACGGCGCCCAGT	618	0,0000	0,0000	0,0000	0,0108
Lead 619	CCCATGTTGGGTCGCCAACCGGCAGTAGTG	619	0,0000	0,0000	0,0000	0,0108
Lead 620	CCCCTGCGACGATTTCTGTTCGATGCTAC	620	0,0000	0,0000	0,0000	0,0108
Lead 621	CCGACGCACGCAACTTGGGTCGCCAAGTAT	621	0,0000	0,0000	0,0000	0,0108
Lead 622	CGACCGTTACGAACCCCCAGAGTGTCGCTG	622	0,0000	0,0000	0,0042	0,0108
Lead 623	CGCACACTTGTGATTCTGAGCCGGTCGAGT	623	0,0000	0,0000	0,0011	0,0108
Lead 624	CGCACCCGTCATTCTGAGCCGTGCACGTGT	624	0,0000	0,0000	0,0000	0,0108
Lead 625	CGCAGATGACTCGTCGGTGTCGTCTCGCCC	625	0,0000	0,0000	0,0000	0,0108
Lead 626	CGCCACTGGACCATTGGGTCGCCAATGGTG	626	0,0000	0,0000	0,0011	0,0108
Lead 627	CGTGCGCACAGGATGTCGATTGGGTCGCCA	627	0,0000	0,0000	0,0011	0,0108
Lead 628	CTCCGCGTTGCGCAGCTGTTCTTGACCCCA	628	0,0000	0,0000	0,0000	0,0108
Lead 629	CTCGTCGCGAAGATTTCGCCCGTTCCGTG	629	0,0000	0,0000	0,0000	0,0108
Lead 630	CTGGATGCCTGCTACCTGATTGGGTCGCCA	630	0,0000	0,0000	0,0011	0,0108
Lead 631	GACATTTCCTGGACTCAGTGTCACGACGAC	631	0,0000	0,0000	0,0011	0,0108
Lead 632	GACGCATCGTATGTATGAACCGTCCCGAGT	632	0,0000	0,0000	0,0000	0,0108
Lead 633	GAGATACTTTTGGGTCGCCACCCTTCGGGC	633	0,0000	0,0000	0,0000	0,0108
Lead 634	GAGGTTTGTGAGCCGTACCACTATACGCGT	634	0,0000	0,0000	0,0000	0,0108
Lead 635	GAGTTGTTACGGCGCTAGACTGCCGCTCCC	635	0,0000	0,0000	0,0021	0,0108
Lead 636	GATGTGCGGAATTCTCGCCCGCTGTCGTG	636	0,0000	0,0000	0,0021	0,0108
Lead 637	GCAGTCTGGGTCGCCAGGCTAGGAATTACT	637	0,0000	0,0000	0,0000	0,0108
Lead 638	GCCACGAACGGACACAGTGCCCGTATCCCC	638	0,0000	0,0000	0,0000	0,0108
Lead 639	GCCAGTCCCAGAGCAAGTAGCTGGGTCTCC	639	0,0000	0,0000	0,0000	0,0108
Lead 640	GCCCGTAACGACATCACTCGGCGTTTCCCC	640	0,0000	0,0000	0,0000	0,0108
Lead 641	GCCCGTCCGCAGCACCTCGACCTGCGTCCA	641	0,0000	0,0000	0,0000	0,0108
Lead 642	GCGGATATGACGTCCCGTCTCCGTAGGTTC	642	0,0000	0,0000	0,0011	0,0108
Lead 643	GGCCCTCCTGGGTTTGACTTGGGTCGCCAC	643	0,0000	0,0000	0,0000	0,0108
Lead 644	GGGACTAGCCGGGCAGCTTCTCCGAGTGTA	644	0,0000	0,0000	0,0011	0,0108
Lead 645	GGTATGGGTCGCCATAGTCCGCGGAGTAGT	645	0,0000	0,0000	0,0000	0,0108
Lead 646	GTCCGGTCATCTCGCGTTGGGTCGCCACAC	646	0,0000	0,0000	0,0000	0,0108
Lead 647	GTTTGGCGTAGGCATGCCGTACGCCTGTTC	647	0,0000	0,0000	0,0000	0,0108
Lead 648	TAGCGATTCCTGGCCCCGGTGGGGTGATGTA	648	0,0000	0,0000	0,0000	0,0108
Lead 649	TCTGGGTCGCCAGGAGCAGTCTCGTCTCGC	649	0,0000	0,0000	0,0000	0,0108

Supplementary Table 4. Multiple alignment of the lead sequences for the Top 200 clusters. Dark rectangles drawn are representing the common motif present in several lead sequences. Lead sequence coloured in light pink are clusters selected for the binding tests.

Name	1	2	3	4	5	6	7	8	9	10	11	12	13	14	15	16	17	18	19	20	21	22	23	24	25	26	27	28	29	30	31	32	33	34	35	36	37	38	39	40	41	42	43	44	45	46	47	48	49	50
Lead 0	-	-	-	-	-	-	-	-	-	-	-	G	C	A	T	T	A	C	G	C	A	G	C	G	C	T	G	C	G	C	A	G	C	G	A	G	T	A	C	C	G	-	-	-	-	-	-	-	-	-
Lead 11	-	-	-	-	-	-	-	-	-	-	G	C	G	G	G	A	C	T	C	T	G	C	C	G	A	A	C	C	G	G	T	C	C	G	T	G	T	G	T	C	-	-	-	-	-	-	-	-	-	-
Lead 58	-	-	-	-	-	-	-	-	-	-	G	A	G	A	G	C	T	C	T	T	A	C	C	G	A	C	C	C	G	C	T	A	C	G	T	G	G	C	T	C	-	-	-	-	-	-	-	-	-	-
Lead 53	-	-	-	-	-	-	-	-	-	-	G	A	A	T	C	T	C	C	G	T	C	C	C	G	C	C	C	G	G	C	T	G	C	G	G	G	T	C	C	C	-	-	-	-	-	-	-	-	-	-
Lead 186	-	-	-	-	-	-	-	-	-	-	G	A	A	T	T	T	T	G	G	T	G	G	C	G	A	A	C	G	G	C	T	C	C	C	A	G	T	G	T	C	-	-	-	-	-	-	-	-	-	-
Lead 99	-	-	-	-	-	-	-	-	-	-	T	G	G	T	C	T	G	C	T	G	G	T	A	G	G	A	C	C	G	C	T	C	C	G	A	G	T	G	C	C	-	-	-	-	-	-	-	-	-	-
Lead 106	-	-	-	-	-	-	-	-	-	-	-	-	-	A	G	T	G	G	T	C	G	T	C	G	G	T	C	C	G	C	T	G	C	G	G	G	T	G	T	C	C	G	T	-	-	-	-	-	-	-
Lead 19	-	-	-	-	-	-	-	-	-	-	G	C	T	G	T	G	A	C	T	G	A	T	G	G	A	C	C	A	G	G	G	C	T	G	A	G	T	G	C	C	-	-	-	-	-	-	-	-	-	-
Lead 174	-	-	-	-	-	-	-	-	-	-	G	A	T	G	T	C	G	C	T	C	C	A	G	T	C	G	T	C	G	G	T	C	T	G	A	G	C	G	T	C	-	-	-	-	-	-	-	-	-	-
Lead 30	-	-	-	-	-	-	-	-	-	-	-	-	-	-	-	-	A	C	G	T	T	C	T	C	G	A	C	C	G	G	T	C	C	G	A	G	T	G	C	G	G	G	A	T	A	C	-	-	-	-
Lead 132	-	-	-	-	-	-	-	A	A	G	T	C	C	C	C	A	T	C	G	T	T	C	T	G	G	A	C	C	G	A	C	C	C	G	A	G	T	-	-	-	-	-	-	-	-	-	-	-	-	-
Lead 137	-	-	-	-	-	-	-	-	-	-	-	G	C	T	C	A	C	T	G	C	T	A	T	G	G	A	C	C	G	C	C	A	C	G	A	G	T	G	C	C	A	-	-	-	-	-	-	-	-	-
Lead 163	-	-	-	-	-	-	-	-	-	-	-	-	-	-	-	-	-	-	-	-	T	A	G	T	G	A	C	C	G	C	T	C	C	G	A	G	T	G	C	A	C	G	G	G	C	A	G	G	G	T
Lead 43	-	-	-	-	-	-	-	-	-	-	G	A	A	T	C	G	A	C	T	T	G	A	T	T	G	G	C	C	G	A	C	A	C	G	A	G	T	G	C	A	-	-	-	-	-	-	-	-	-	-
Lead 153	-	-	-	-	-	-	-	-	-	-	G	A	A	G	G	G	T	T	C	T	G	G	T	T	G	G	C	C	G	T	C	T	C	G	A	G	T	G	T	A	-	-	-	-	-	-	-	-	-	-
Lead 33	-	-	-	-	-	-	-	C	C	C	C	C	C	C	T	A	G	T	G	T	T	C	T	G	A	G	C	C	G	A	A	C	C	G	A	G	T	-	-	-	-	-	-	-	-	-	-	-	-	-
Lead 51	-	-	-	-	-	-	-	G	C	T	C	G	C	T	C	C	G	T	T	T	T	C	T	G	G	G	C	C	G	C	T	C	C	G	A	G	T	-	-	-	-	-	-	-	-	-	-	-	-	-
Lead 98	-	-	-	-	-	-	-	G	A	A	T	G	C	C	T	A	T	G	T	T	T	G	T	G	A	G	C	C	G	A	T	C	C	G	A	G	T	-	-	-	-	-	-	-	-	-	-	-	-	-
Lead 38	-	-	-	-	-	-	-	A	G	C	G	G	C	A	T	A	T	G	T	T	T	C	T	G	A	G	C	G	G	G	C	C	C	C	A	G	T	-	-	-	-	-	-	-	-	-	-	-	-	-
Lead 66	-	-	-	-	-	-	-	C	A	C	C	T	C	G	G	A	T	A	T	T	G	C	T	G	A	G	C	G	G	A	T	C	C	C	A	G	T	-	-	-	-	-	-	-	-	-	-	-	-	-
Lead 37	-	-	-	-	-	-	-	A	C	T	G	C	G	G	G	T	A	A	T	T	C	C	T	G	A	G	C	C	G	C	T	C	C	G	A	G	T	-	-	-	-	-	-	-	-	-	-	-	-	-
Lead 91	-	-	-	-	-	-	-	G	A	T	G	C	C	G	T	G	T	C	T	G	T	C	T	G	A	G	C	C	G	C	T	G	C	G	A	G	T	-	-	-	-	-	-	-	-	-	-	-	-	-
Lead 185	-	-	-	-	-	-	-	C	T	A	G	T	G	C	A	A	T	T	A	T	C	C	T	G	A	G	C	C	G	C	G	C	C	G	A	G	T	-	-	-	-	-	-	-	-	-	-	-	-	-
Lead 52	-	-	-	-	-	-	-	-	-	-	G	A	C	C	C	G	G	T	T	C	T	G	C	T	A	G	C	G	G	T	C	C	C	C	A	G	T	G	T	A	-	-	-	-	-	-	-	-	-	-
Lead 60	-	-	-	-	-	-	-	G	C	A	G	T	C	C	C	T	G	T	T	T	C	C	T	G	A	G	C	G	G	T	C	C	C	C	A	G	T	-	-	-	-	-	-	-	-	-	-	-	-	-
Lead 54	-	-	-	-	-	-	-	G	G	A	G	A	G	C	G	A	T	A	T	T	G	C	T	G	A	G	C	C	A	T	G	T	T	G	C	G	T	-	-	-	-	-	-	-	-	-	-	-	-	-
Lead 183	-	-	-	-	-	-	-	A	G	A	G	C	C	C	G	A	T	A	T	T	C	T	C	G	A	A	C	C	G	T	T	C	C	G	A	G	T	-	-	-	-	-	-	-	-	-	-	-	-	-
Lead 148	-	-	-	-	-	-	-	G	A	G	A	G	T	A	G	T	T	G	T	T	T	C	T	G	G	A	C	G	G	G	T	C	C	C	A	G	T	-	-	-	-	-	-	-	-	-	-	-	-	-
Lead 112	-	-	-	-	-	-	-	-	-	C	A	G	A	C	C	G	C	A	C	T	T	C	T	G	A	G	C	C	G	A	T	C	T	T	C	G	A	G	T	-	-	-	-	-	-	-	-	-	-	-
Lead 192	-	-	-	-	-	-	-	-	-	G	A	C	G	C	C	A	A	T	C	T	T	G	T	G	A	G	C	C	G	G	C	A	C	T	C	G	A	G	T	-	-	-	-	-	-	-	-	-	-	-
Lead 164	-	-	-	-	-	-	-	-	-	A	A	T	G	C	G	G	A	T	G	C	T	G	G	A	C	A	C	C	G	A	G	T	C	G	A	G	T	C	T	-	-	-	-	-	-	-	-	-	-	-
Lead 199	-	-	-	-	-	-	-	-	-	-	-	-	-	G	G	A	C	T	G	A	T	G	G	A	C	A	C	C	G	T	C	G	C	G	A	G	T	C	T	G	T	C	T	-	-	-	-	-	-	-
Lead 170	-	-	-	-	-	-	-	-	-	A	A	C	T	G	G	G	G	C	T	C	T	G	G	A	C	A	C	G	G	T	T	C	C	C	A	G	T	C	T	-	-	-	-	-	-	-	-	-	-	-
Lead 63	-	-	-	-	-	-	-	-	-	-	-	-	-	-	A	G	A	T	T	T	C	A	T	T	A	C	C	G	G	C	C	C	A	C	C	C	A	G	G	C	A	C	G	A	-	-	-	-	-	-
Lead 135	-	-	-	-	-	-	-	-	-	-	-	-	-	G	A	G	G	T	T	T	C	T	C	G	A	G	C	G	G	C	A	C	T	A	G	G	T	G	C	C	A	G	T	-	-	-	-	-	-	-
Lead 169	-	-	-	-	-	-	-	T	A	C	C	C	C	G	A	G	A	T	T	T	C	T	T	G	A	G	C	G	G	C	A	G	C	C	A	G	T	-	-	-	-	-	-	-	-	-	-	-	-	-
Lead 2	-	-	-	-	-	-	-	-	-	-	A	A	T	C	G	G	A	A	T	T	C	C	T	G	A	G	C	C	G	T	G	C	C	T	C	C	G	A	G	T	-	-	-	-	-	-	-	-	-	-
Lead 108	-	-	-	-	-	-	-	-	-	-	G	A	C	G	C	A	A	A	T	T	C	G	T	G	A	G	C	C	G	C	G	G	T	A	G	C	G	T	G	T	-	-	-	-	-	-	-	-	-	-
Lead 180	-	-	-	-	-	-	-	-	-	-	C	A	C	G	G	A	A	T	T	T	C	T	G	T	A	G	C	C	G	A	G	C	A	T	T	C	G	T	G	G	-	-	-	-	-	-	-	-	-	-
Lead 64	-	-	-	-	-	-	-	-	-	-	C	C	A	C	G	A	A	G	T	T	T	C	G	G	A	G	C	G	G	T	C	C	C	A	T	C	C	T	G	T	-	-	-	-	-	-	-	-	-	-
Lead 26	-	-	-	-	-	-	-	-	-	-	-	-	-	G	G	C	A	A	T	T	G	G	T	G	A	G	C	A	G	A	G	A	C	G	C	C	C	T	C	G	A	G	T	-	-	-	-	-	-	-
Lead 69	-	-	-	-	-	-	-	-	-	-	-	-	-	A	G	G	A	A	T	T	G	G	G	G	A	G	C	C	G	G	C	G	A	T	C	C	G	T	C	G	A	G	T	-	-	-	-	-	-	-
Lead 162	-	-	-	-	-	-	-	-	-	-	-	-	-	G	A	A	T	A	T	T	G	C	A	G	A	G	C	C	G	G	C	C	A	G	A	T	C	C	C	G	A	G	T	-	-	-	-	-	-	-
Lead 124	-	-	-	-	-	-	-	-	-	-	-	-	-	A	A	A	T	T	A	T	C	C	T	G	G	G	C	C	G	A	G	G	A	G	T	C	C	T	C	G	A	G	T	-	-	-	-	-	-	-
Lead 3	-	-	-	-	-	-	-	-	-	-	-	-	G	G	A	A	T	T	G	G	G	C	T	G	A	G	C	C	G	A	G	G	T	C	T	C	T	C	G	A	G	T	-	-	-	-	-	-	-	-
Lead 21	-	-	-	-	-	-	-	-	-	-	-	-	G	A	G	A	C	A	T	T	T	C	T	G	G	G	C	C	G	G	G	T	C	T	T	G	C	C	G	T	G	T	-	-	-	-	-	-	-	-
Lead 59	-	-	-	-	-	-	-	-	-	-	-	-	G	A	G	A	T	C	T	G	T	C	T	G	A	G	C	C	G	G	A	A	G	C	T	A	C	C	G	T	G	T	-	-	-	-	-	-	-	-
Lead 76	-	-	-	-	-	-	-	-	-	-	-	-	G	A	G	C	A	T	T	T	C	A	T	G	A	G	C	C	G	C	T	A	T	C	G	G	G	C	G	A	G	T	-	-	-	-	-	-	-	-
Lead 103	-	-	-	-	-	-	-	-	-	-	-	-	G	C	T	A	A	T	T	C	T	C	T	G	G	G	C	C	G	T	C	G	C	A	G	T	A	C	G	A	G	T	-	-	-	-	-	-	-	-
Lead 20	-	-	-	-	-	-	-	-	-	-	-	-	G	C	A	G	A	A	T	T	T	C	T	G	A	G	C	G	A	G	C	A	C	G	G	G	C	C	C	T	G	T	-	-	-	-	-	-	-	-
Lead 184	-	-	-	-	-	-	-	-	-	-	-	-	-	-	C	A	A	G	T	T	T	C	T	G	A	G	C	G	A	G	T	A	C	C	T	C	C	C	C	T	C	T	G	T	-	-	-	-	-	-
Lead 95	-	-	-	-	-	-	-	-	-	-	-	-	-	-	-	G	C	A	T	T	T	G	T	G	A	G	C	C	G	G	C	A	T	C	T	C	G	C	A	T	C	G	T	G	T	-	-	-	-	-
Lead 16	-	-	-	-	-	-	-	-	-	-	-	-	-	-	-	-	-	-	G	C	T	C	T	G	A	G	C	C	G	A	G	G	G	A	A	T	C	G	T	C	G	C	T	C	G	T	G	T	-	-
Lead 155	-	-	-	-	-	-	-	-	-	-	-	-	-	-	-	-	T	A	G	T	T	C	T	G	A	G	C	C	G	G	T	G	G	A	C	C	C	T	G	G	C	C	G	T	G	T	-	-	-	-
Lead 29	-	-	-	-	-	-	-	-	-	-	-	-	-	-	A	G	C	G	T	T	T	C	G	G	A	G	C	C	G	A	T	G	C	C	C	A	C	T	T	C	G	A	G	T	-	-	-	-	-	-
Lead 44	-	-	-	-	-	-	-	-	-	-	-	-	-	-	G	C	A	T	T	T	C	C	T	G	A	G	C	C	G	A	T	G	C	T	T	C	T	G	C	C	G	A	G	T	-	-	-	-	-	-
Lead 110	-	-	-	-	-	-	-	-	-	-	-	-	-	-	-	-	A	G	A	T	T	C	T	G	A	G	C	C	G	A	T	G	T	T	G	T	A	C	C	C	C	G	A	G	T	-	-	-	-	-
Lead 197	-	-	-	-	-	-	-	-	-	-	-	-	-	-	-	-	-	C	A	G	T	C	T	G	A	G	C	A	G	A	T	G	T	G	G	G	T	C	G	C	C	G	C	T	T	C	T	-	-	-
Lead 5	-	-	-	-	-	-	-	-	-	C	A	A	G	C	G	A	A	G	T	T	G	T	G	C	G	C	C	C	G	C	G	T	C	G	T	G	G	T	C	-	-	-	-	-	-	-	-	-	-	-
Lead 154	-	-	-	-	-	-	G	A	C	C	A	C	C	A	G	G	G	G	A	T	T	T	C	C	G	C	C	C	G	T	T	C	C	G	T	G	-	-	-	-	-	-	-	-	-	-	-	-	-	-
Lead 190	-	-	-	-	-	-	C	A	T	C	T	A	G	T	C	G	C	A	A	T	T	G	T	C	G	C	C	G	G	C	T	C	C	C	T	G	-	-	-	-	-	-	-	-	-	-	-	-	-	-
Lead 73	-	-	-	-	-	-	G	C	C	C	G	A	C	C	A	T	A	T	T	C	T	T	G	G	C	C	C	C	C	G	T	C	G	G	T	G	-	-	-	-	-	-	-	-	-	-	-	-	-	-
Lead 125	-	-	-	-	-	-	G	C	T	C	G	T	C	A	T	G	T	T	C	T	T	G	A	C	C	A	C	G	G	G	T	C	G	G	T	G	-	-	-	-	-	-	-	-	-	-	-	-	-	-
Lead 7	-	-	-	-	-	-	-	-	-	-	-	-	-	A	G	G	A	T	T	T	C	C	T	G	G	A	C	C	T	G	C	G	C	G	C	A	G	G	T	C	A	C	T	-	-	-	-	-	-	-
Lead 68	-	-	-	-	-	-	-	-	-	A	C	G	G	C	G	C	A	T	T	T	C	C	T	G	G	A	C	C	A	A	C	G	G	T	C	A	C	C	T	-	-	-	-	-	-	-	-	-	-	-
Lead 28	-	-	-	-	-	-	-	-	-	-	-	-	-	-	-	G	A	G	T	T	C	C	T	G	G	A	G	C	A	C	G	C	C	T	C	A	C	G	A	C	C	A	T	C	G	-	-	-	-	-
Lead 194	-	-	-	-	-	-	-	-	-	-	-	-	-	-	-	-	G	A	T	T	C	A	T	G	G	A	C	C	G	C	C	G	G	T	C	A	C	T	G	C	A	C	C	C	T	C	-	-	-	-
Lead 22	-	-	-	-	-	-	-	-	-	-	-	-	-	-	-	G	A	T	T	T	C	C	T	G	G	A	G	A	C	T	C	G	T	C	T	C	A	C	T	G	G	C	G	A	C	-	-	-	-	-
Lead 48	-	-	-	-	-	-	-	-	-	-	-	-	-	-	G	A	A	C	T	T	C	C	T	C	G	A	C	C	C	G	C	T	G	G	T	C	A	T	T	G	C	C	A	C	-	-	-	-	-	-
Lead 1	-	-	-	-	-	-	C	A	G	T	G	C	A	G	A	C	G	A	T	G	C	T	G	G	G	T	C	G	C	C	A	G	G	G	A	T	-	-	-	-	-	-	-	-	-	-	-	-	-	-
Lead 122	-	-	-	-	G	T	A	C	G	G	A	C	T	C	A	C	C	C	T	G	C	T	G	G	G	T	C	G	C	C	A	G	A	G	-	-	-	-	-	-	-	-	-	-	-	-	-	-	-	-
Lead 65	-	-	-	-	-	A	A	C	C	A	G	C	C	C	G	A	C	A	C	G	T	T	G	G	G	T	C	G	C	C	A	C	C	G	A	-	-	-	-	-	-	-	-	-	-	-	-	-	-	-
Lead 181	-	-	-	-	-	C	A	C	G	G	G	A	C	C	G	A	C	C	A	G	T	T	G	G	G	T	C	G	C	C	A	G	C	T	A	-	-	-	-	-	-	-	-	-	-	-	-	-	-	-
Lead 13	-	-	-	A	G	C	A	G	C	T	T	A	C	C	T	C	G	G	C	T	C	T	G	G	G	T	C	G	C	C	A	G	G	-	-	-	-	-	-	-	-	-	-	-	-	-	-	-	-	-
Lead 78	-	-	C	A	T	A	C	C	C	A	T	T	C	G	C	T	C	G	A	T	C	T	G	G	G	T	C	G	C	C	A	G	-	-	-	-	-	-	-	-	-	-	-	-	-	-	-	-	-	-
Lead 115	-	C	C	A	T	A	C	G	T	G	C	A	C	G	G	C	C	T	G	A	T	T	G	G	G	T	C	G	C	C	A	-	-	-	-	-	-	-	-	-	-	-	-	-	-	-	-	-	-	-
Lead 23	-	-	-	C	C	G	C	C	A	T	G	C	G	A	C	T	G	A	C	A	T	T	G	G	G	T	C	G	C	C	A	G	G	-	-	-	-	-	-	-	-	-	-	-	-	-	-	-	-	-
Lead 140	-	-	-	T	A	C	C	G	A	C	G	C	A	A	T	T	G	A	C	C	T	T	G	G	G	T	C	G	C	C	A	G	G	-	-	-	-	-	-	-	-	-	-	-	-	-	-	-	-	-
Lead 160	-	-	-	-	-	-	-	C	G	C	G	C	A	A	T	C	G	T	A	G	T	T	G	G	G	T	C	G	C	C	A	G	C	T	C	A	C	-	-	-	-	-	-	-	-	-	-	-	-	-
Lead 72	-	-	-	-	A	T	A	C	T	C	C	G	C	T	A	G	G	C	G	G	T	T	G	G	G	T	C	G	C	C	A	G	C	C	-	-	-	-	-	-	-	-	-	-	-	-	-	-	-	-
Lead 101	-	-	-	-	-	-	-	G	A	C	G	G	C	T	A	C	G	G	G	A	T	T	G	G	G	T	C	G	C	C	A	G	T	C	C	C	C	-	-	-	-	-	-	-	-	-	-	-	-	-
Lead 189	-	-	-	-	-	-	-	C	A	C	C	G	G	A	C	A	G	G	G	G	T	T	G	G	G	T	C	G	C	C	A	A	T	A	C	T	T	-	-	-	-	-	-	-	-	-	-	-	-	-
Lead 90	-	-	C	A	G	T	G	C	T	C	G	T	A	T	C	G	C	A	A	C	T	T	G	G	G	T	C	G	C	C	A	G	-	-	-	-	-	-	-	-	-	-	-	-	-	-	-	-	-	-
Lead 142	-	-	G	A	G	C	G	C	T	C	T	C	T	C	T	T	C	T	G	C	T	T	G	G	G	T	C	G	C	C	A	G	-	-	-	-	-	-	-	-	-	-	-	-	-	-	-	-	-	-
Lead 114	-	-	-	-	-	C	A	C	T	C	C	C	G	C	T	G	A	C	C	T	A	T	G	G	G	T	C	G	C	C	A	G	C	A	G	-	-	-	-	-	-	-	-	-	-	-	-	-	-	-
Lead 167	-	-	-	-	C	C	G	G	T	A	C	T	T	C	T	G	G	C	A	G	T	T	G	G	G	T	C	G	C	C	A	G	C	C	-	-	-	-	-	-	-	-	-	-	-	-	-	-	-	-
Lead 39	-	-	-	-	-	-	G	G	C	G	C	A	C	G	A	A	T	C	G	C	T	T	G	G	G	T	C	G	C	C	A	G	G	C	G	A	-	-	-	-	-	-	-	-	-	-	-	-	-	-
Lead 77	-	-	-	-	-	-	-	-	-	-	C	A	G	G	A	T	C	G	C	T	T	G	G	G	T	C	G	C	C	A	G	C	G	G	G	C	C	A	A	G	-	-	-	-	-	-	-	-	-	-
Lead 123	-	-	-	-	-	-	-	-	-	G	T	C	G	A	C	G	A	G	C	T	T	G	G	G	T	C	G	C	C	A	G	C	C	G	G	C	A	G	A	-	-	-	-	-	-	-	-	-	-	-
Lead 161	-	-	-	G	A	A	C	T	C	A	C	G	C	A	T	C	G	G	G	C	T	T	G	G	G	T	C	G	C	C	A	C	A	-	-	-	-	-	-	-	-	-	-	-	-	-	-	-	-	-
Lead 187	-	-	-	T	A	T	C	T	C	C	C	T	C	C	G	C	T	G	A	C	T	T	G	G	G	T	C	G	C	C	A	C	T	-	-	-	-	-	-	-	-	-	-	-	-	-	-	-	-	-
Lead 179	-	-	-	A	G	T	T	G	G	G	C	T	C	T	A	G	A	C	T	C	G	T	G	G	G	T	C	G	C	C	A	G	A	-	-	-	-	-	-	-	-	-	-	-	-	-	-	-	-	-
Lead 4	-	-	-	-	-	-	-	-	-	-	-	-	-	C	A	C	G	G	A	T	C	T	G	G	G	T	C	G	C	C	A	G	G	G	C	C	G	C	A	T	C	C	T	-	-	-	-	-	-	-
Lead 107	-	-	-	-	-	-	-	-	-	-	-	-	-	-	T	A	G	G	A	T	C	T	G	G	G	T	C	G	C	C	A	C	G	G	T	C	C	T	C	C	C	C	C	G	-	-	-	-	-	-
Lead 8	-	-	-	-	-	-	-	-	-	-	-	-	-	-	A	G	C	G	G	T	C	T	G	G	G	T	C	G	C	C	A	G	A	A	C	T	C	G	C	A	C	G	T	G	-	-	-	-	-	-
Lead 80	-	-	-	-	-	-	-	-	-	-	-	-	-	-	T	G	G	G	G	T	C	T	G	G	G	T	C	G	C	C	A	G	A	G	G	C	C	G	C	C	A	G	T	G	-	-	-	-	-	-

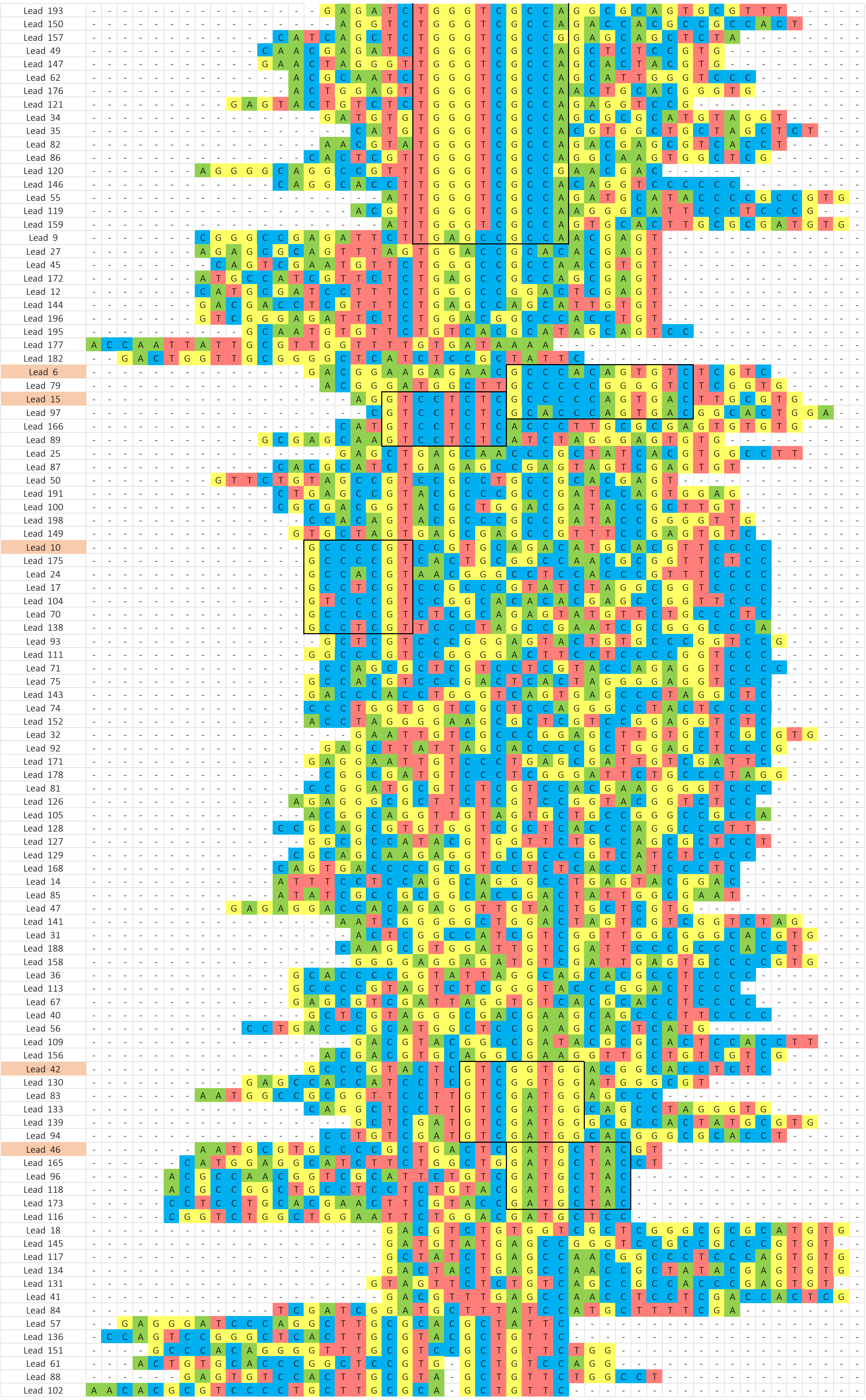
Lead 193
Lead 150
Lead 157
Lead 49
Lead 147
Lead 62
Lead 176
Lead 121
Lead 34
Lead 35
Lead 82
Lead 86
Lead 120
Lead 146
Lead 55
Lead 119
Lead 159
Lead 9
Lead 27
Lead 45
Lead 172
Lead 12
Lead 144
Lead 196
Lead 195
Lead 177
Lead 182
Lead 6
Lead 79
Lead 15
Lead 97
Lead 166
Lead 89
Lead 25
Lead 87
Lead 50
Lead 191
Lead 100
Lead 198
Lead 149
Lead 10
Lead 175
Lead 24
Lead 17
Lead 104
Lead 70
Lead 138
Lead 93
Lead 111
Lead 71
Lead 75
Lead 143
Lead 74
Lead 152
Lead 32
Lead 92
Lead 171
Lead 178
Lead 81
Lead 126
Lead 105
Lead 128
Lead 127
Lead 129
Lead 168
Lead 14
Lead 85
Lead 47
Lead 141
Lead 31
Lead 188
Lead 158
Lead 36
Lead 113
Lead 67
Lead 40
Lead 56
Lead 109
Lead 156
Lead 42
Lead 130
Lead 83
Lead 133
Lead 139
Lead 94
Lead 46
Lead 165
Lead 96
Lead 118
Lead 173
Lead 116
Lead 18
Lead 145
Lead 117
Lead 134
Lead 131
Lead 41
Lead 84
Lead 57
Lead 136
Lead 151
Lead 61
Lead 88
Lead 102

6. Additional binding experiment

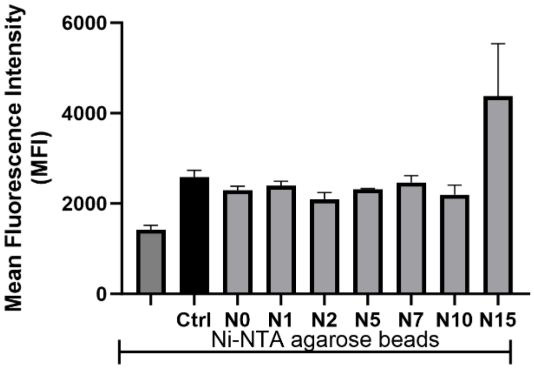

Supplementary Figure 7. Flow cytometry binding analysis of all aptamer candidates with Ni-NTA agarose beads. 10 nM 5'- FAM-labelled sequences along with a control sequence (Ctrl) were incubated with Ni-NTA agarose beads for 30 minutes and analyzed by flow cytometry using the Attune NxT Flow Cytometer.

7. Characterization of compounds

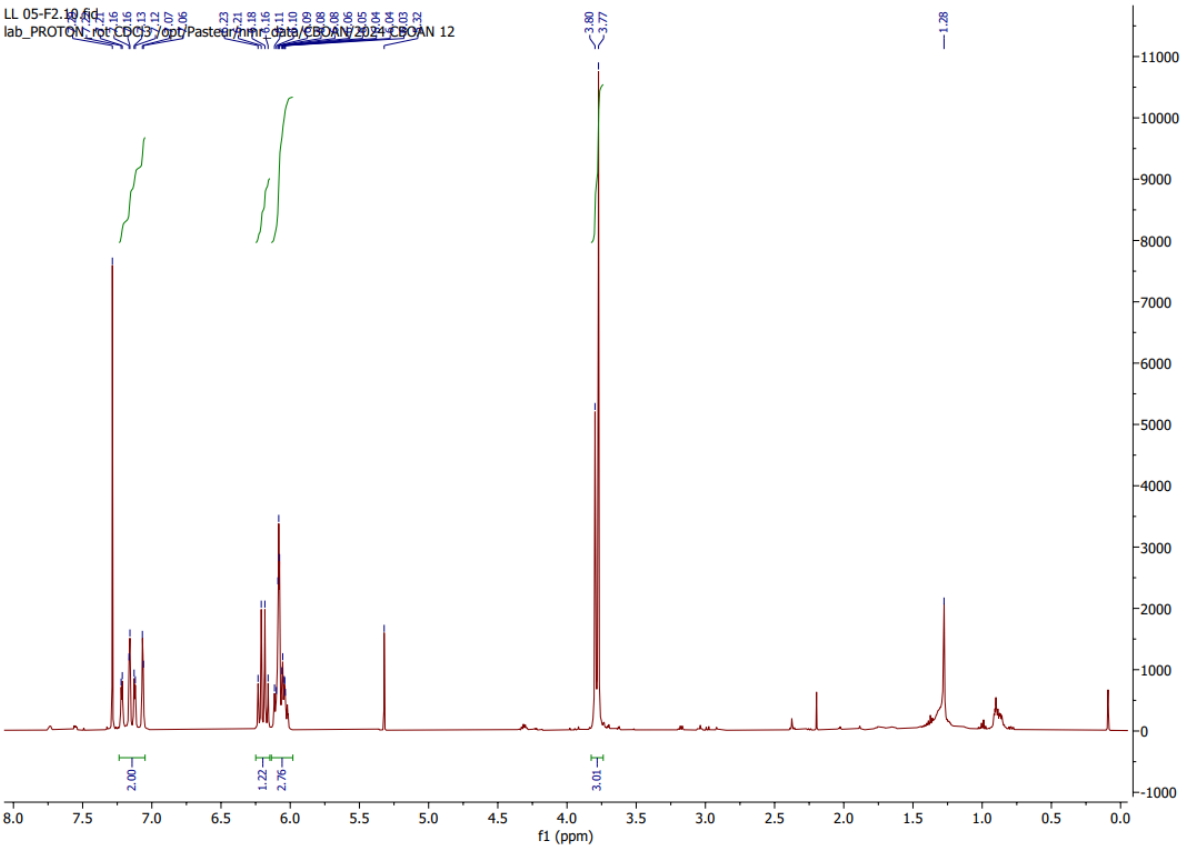

Supplementary Figure 8. ^{1}H NMR (500 MHz, $CDCl_3$) spectrum of compound **5**.

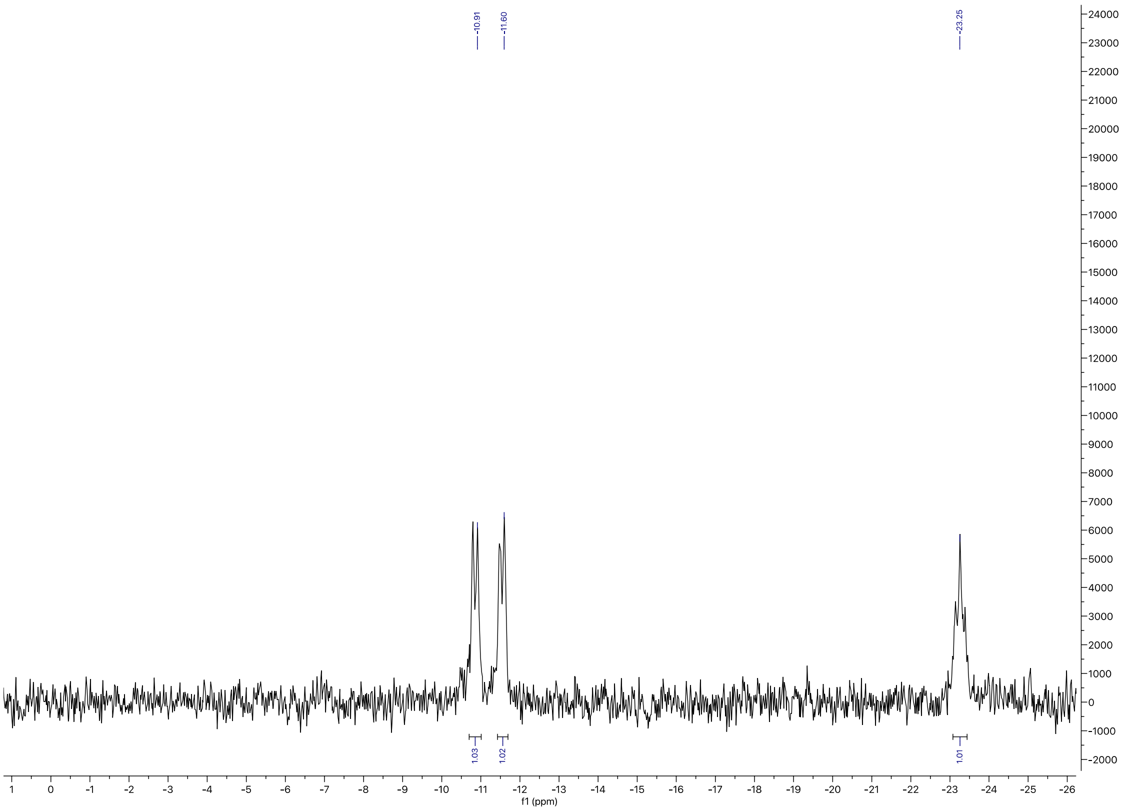

Supplementary Figure 9. ^{31}P-NMR (162 MHz, D_2O) of **dU^{COTc}TP 6**.

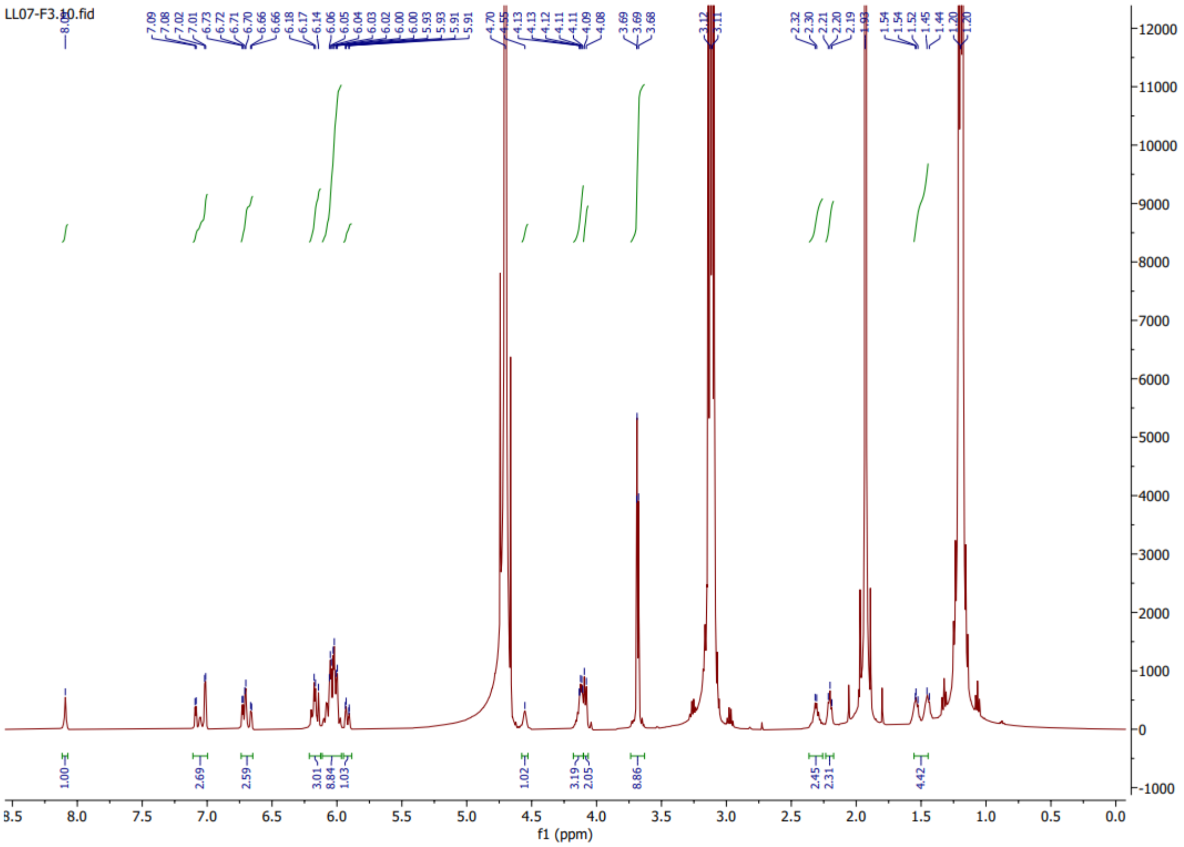

Supplementary Figure 10. ^{1}H-NMR (400 MHz, D_2O) of **dU^{COTc}TP 6**.

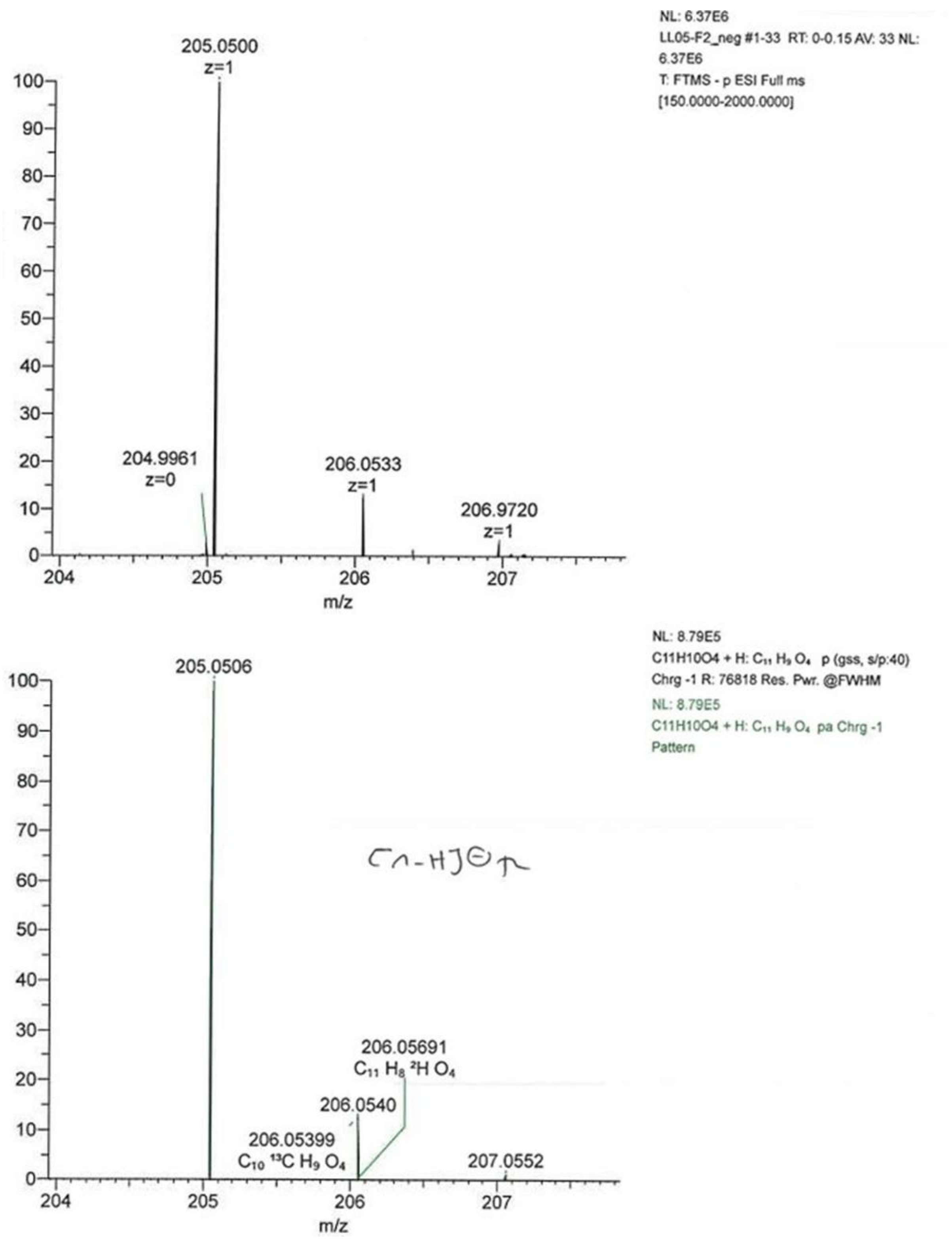

Supplementary Figure 11. HRMS analysis of compound **5**.

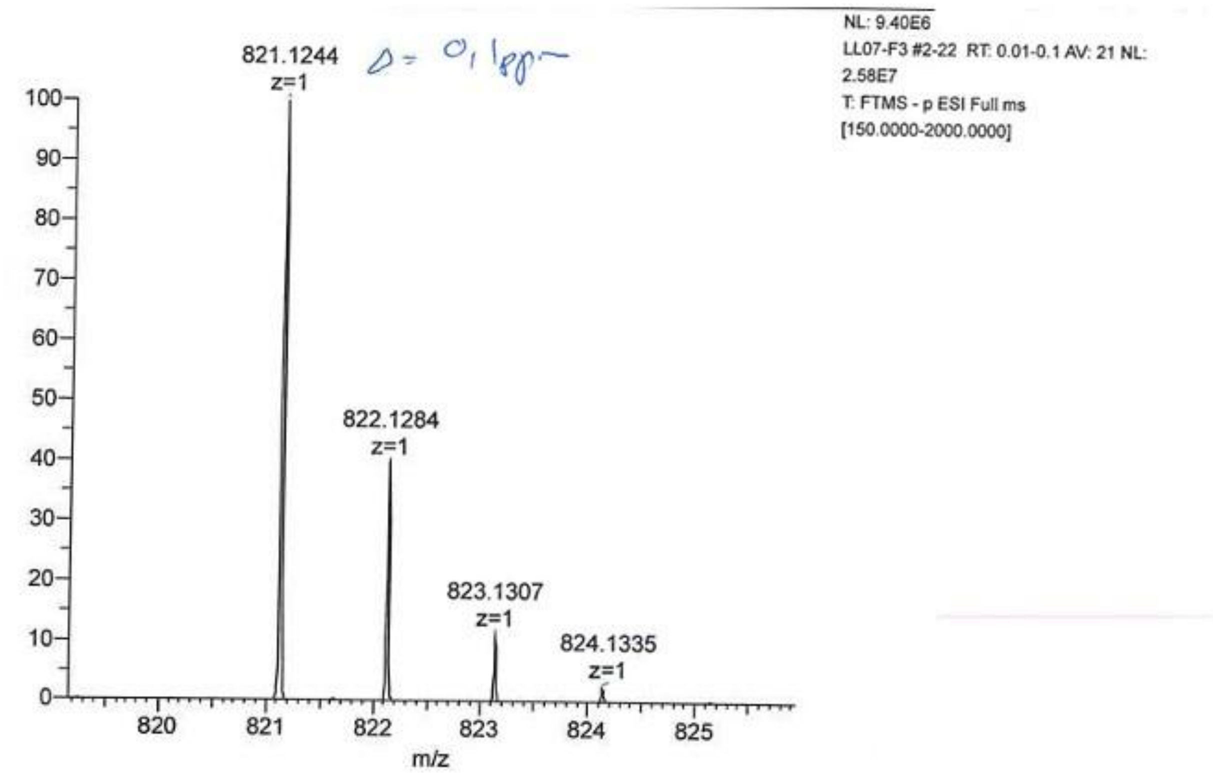

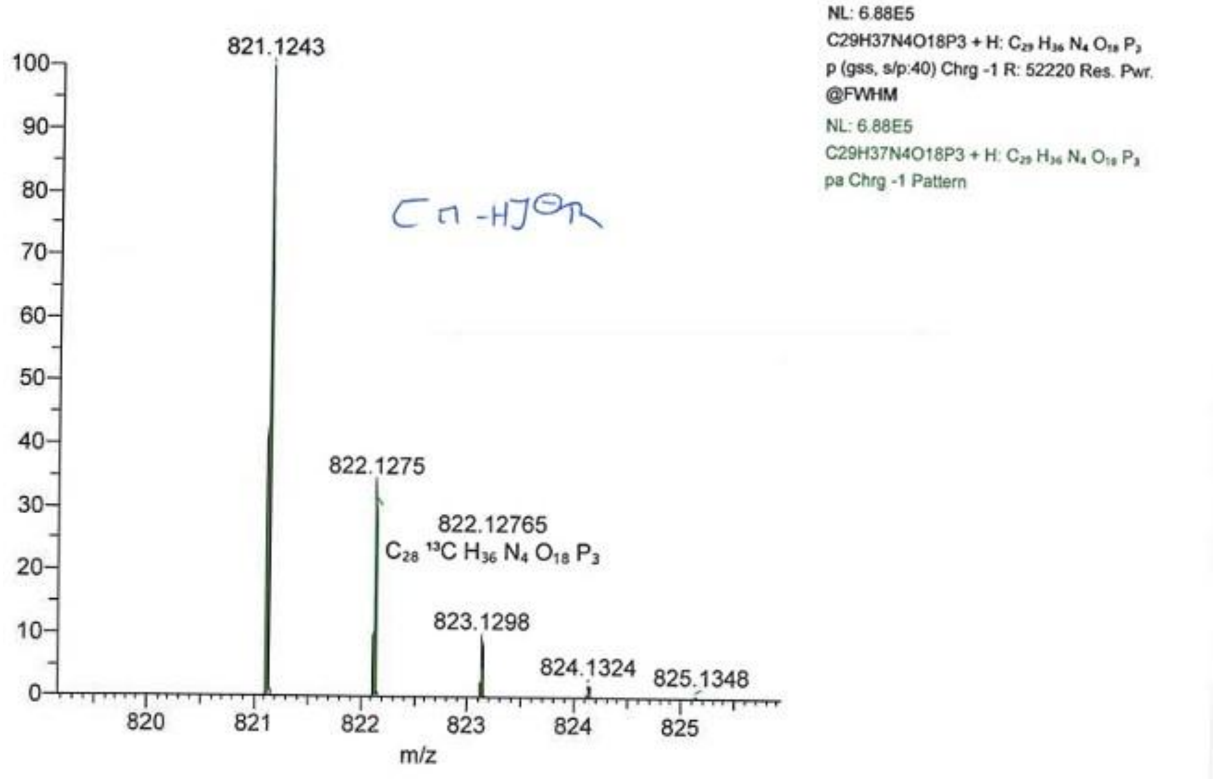

Supplementary Figure 12. HRMS analysis of compound **dU^{COTc}TP 6**.

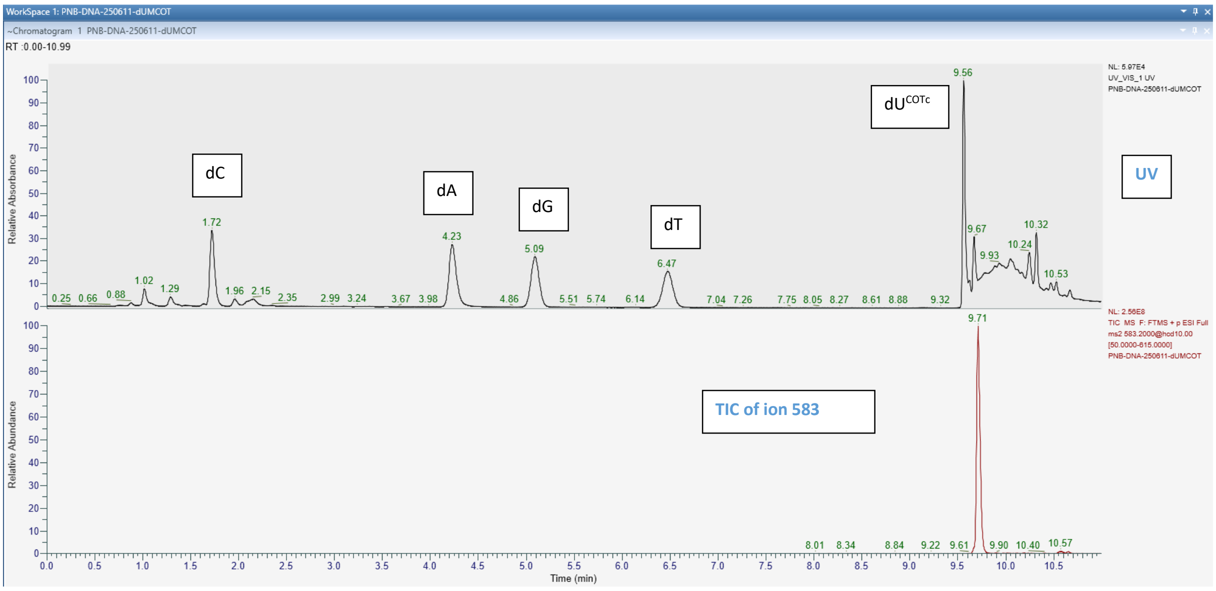

Supplementary Figure 13. LC-MS digestion chromatogram monitored by UV detection showing the nucleoside digestion profile of dsDNA after PEX reaction with template **T3** and primer **P5**. The chromatogram reveals five distinct peaks, four of which correspond to the canonical nucleosides of the template and primer: deoxycytidine (dC), deoxyadenine (dA), deoxyguanine (dG) and deoxythymidine (dT). The additional peak represents the modified deoxyuridine **dU^{COTc} 6**.

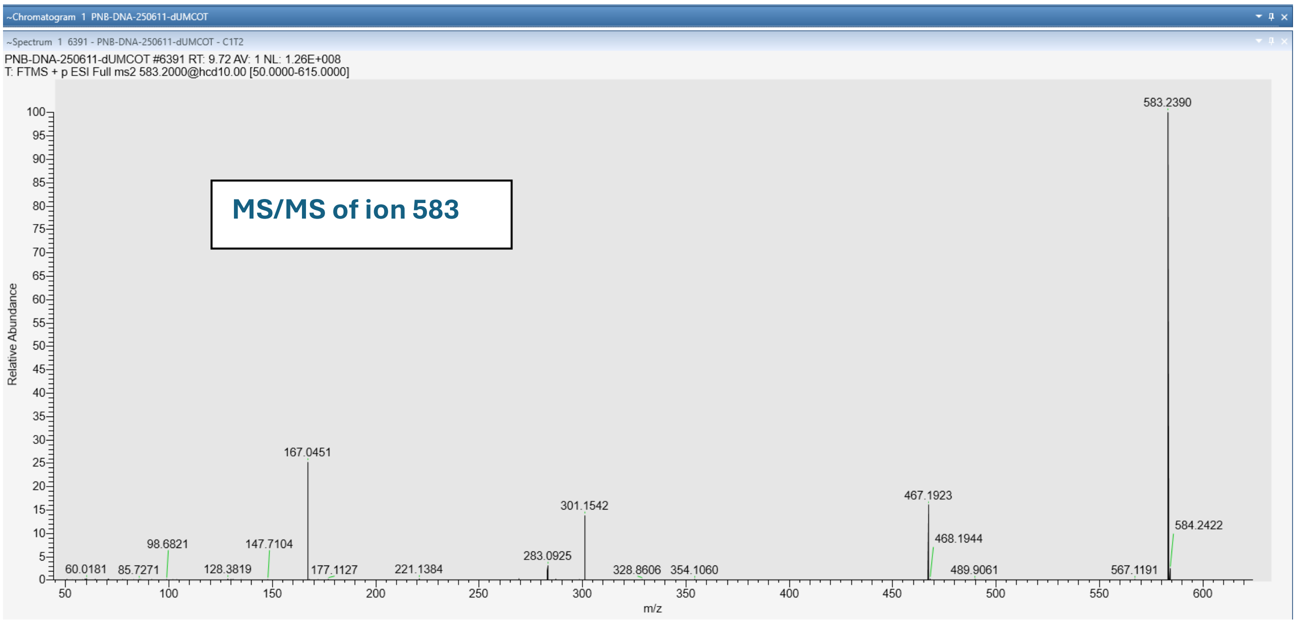

Supplementary Figure 14. MS/MS spectrum after digestion experiment.

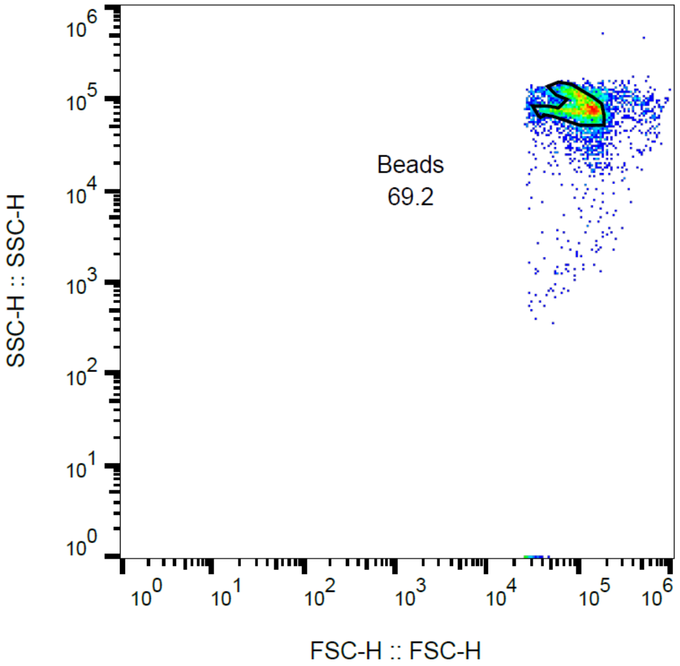

Supplementary Figure 15. Representative example of initial gating with empty beads.
